# Social Relationship as a Factor for the Development of Stress Incubation in Adult Mice

**DOI:** 10.3389/fnbeh.2022.854486

**Published:** 2022-05-24

**Authors:** Ray X. Lee, Greg J. Stephens, Bernd Kuhn

**Affiliations:** ^1^Optical Neuroimaging Unit, Okinawa Institute of Science and Technology (OIST) Graduate University, Okinawa, Japan; ^2^Biological Physics Theory Unit, Okinawa Institute of Science and Technology (OIST) Graduate University, Okinawa, Japan; ^3^Department of Physics and Astronomy, Vrije Universiteit Amsterdam, Amsterdam, Netherlands

**Keywords:** PTSD, stress, animal emotionality, social bonding, animal disease model, behavioral test, behavioral analysis, anterior cingulate cortex

## Abstract

While stress reactions can emerge long after the triggering event, it remains elusive how they emerge after a protracted, seemingly stress-free period during which stress incubates. Here, we study the behavioral development in mice isolated after observing an aggressive encounter inflicted upon their pair-housed partners. We developed a spatially resolved fine-scale behavioral analysis and applied it to standard behavioral tests. It reveals that the seemingly sudden behavioral changes developed gradually. These behavioral changes were not observed if the aggressive encounter happened to a stranger mouse, suggesting that social bonding is a prerequisite for stress incubation in this paradigm. This finding was corroborated by hemisphere-specific morphological changes in cortex regions centering at the anterior cingulate cortex, a cognitive and emotional center. Our non-invasive analytical methods to capture informative behavioral details may have applications beyond laboratory animals.

## Introduction

Stress incubation describes the time interval following an aversive event during which stress reactions emerge or increase (Bebbington et al., 1993). The phenomenon of stress incubation has received serious attention in human psychiatry due to its dramatic impact of subsequent symptoms on human wellbeing, such as post-traumatic stress disorder (PTSD) (DSM-III, 1980; DSM-5, 2013) that shows a wide variety and surprising inconsistency of symptoms and a highly variable delay of onset after a protracted symptom-free period (Andrews et al., 2007; Pai et al., 2017).

A delay period before showing substantial stress reactions, suggesting stress incubation, was reported in the context of rodent models simulating impacts of aversive experience (Davis, 1989; Pamplona et al., 2011; Warren et al., 2013; Tsuda et al., 2015; Sillivan et al., 2017). The wide variety of aversive stimuli in these models range from acute physical stress (Balogh et al., 2002; Philbert et al., 2011) to prolonged witnessing of social defeat (Warren et al., 2013). However, in the previously reported paradigms of witnessing stress, an over-a-week prolonged period of repeated aversive stimuli overlapped stress inductions with stress incubation, which challenges the identification of potential behavioral signatures at the beginning of stress incubation. Previously reported paradigms of acute physical stress, on the other hand, are challenged by the behavioral consequences that may simply resulted from the prolonged development of physical injuries and by inferring from a single, focused behavioral metric, mostly the length of freezing behavior.

**FIGURE 1:**
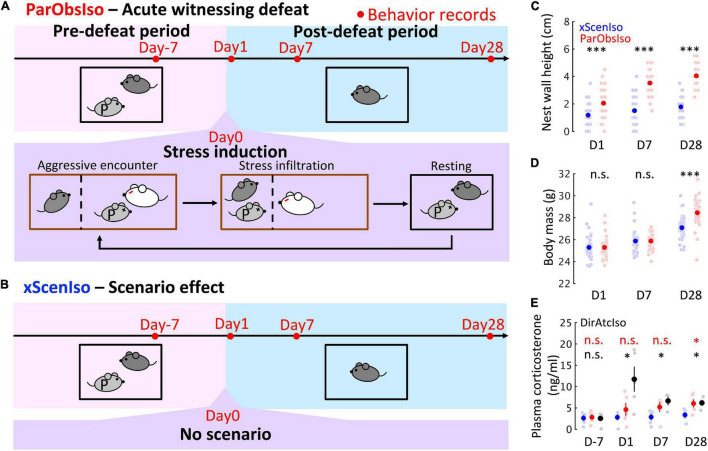
Paradigm inducing stress incubation in mice with long-term and delayed effects on behaviors and physical conditions. **(A)** Paradigm with acute psychosocial exposure to witnessing stress in mice. Focal mouse [dark gray; Partner-Observing-Isolated (ParObsIso) mouse], partner mouse (light gray P), aggressor mouse (white), focal mouse’s homecage (black), aggressor’s homecage (brown), and wire-meshed divider (dashed line). **(B)** No-Scenario-Isolated (xScenIso) mice were separated without exposure to witnessing stress and identified the scenario effect in the behavioral paradigm. **(C)** Nest wall heights show long-lasting significant differences after exposure to witnessing stress (*n*_*xScenIso*_ = 47, *n*_*ParObsIso*_ = 47). **(D)** Body mass shows a significant increase 28 days after exposure to witnessing stress (*n*_*xScenIso*_ = 47, *n*_*ParObsIso*_ = 47). **(E)** Baseline plasma corticosterone level increased after exposure to witnessing stress for both ParObsIso mice and their partners, the DirAtcIso mice (*n*_*xScenIso*_ = 5, *n*_*ParObsIso*_ = 5, $n_{D\,i\,r\,A\,t\,c\,I\,s\,o}^{D-7,D\,1}$ = 5, $n_{D\,i\,r\,A\,t\,c\,I\,s\,o}^{D\,7,D\,14}$ = 3). Error bars indicate standard errors of the means; n.s., *p* ≥ 0.05; *, 0.01 ≤ *p* < 0.05; ^***^p < 0.001; Tukey’s range test.

To address the limitations of previous animal studies of stress incubation, we systematically and quantitatively examined multiple physiological conditions (body mass, corticosterone level, brain connectome), spontaneous behaviors (nest-building, light-dark box, open field, locomotion), and social interactions (female strangers, male strangers, pair-housed partners) of mice after observing acute social stress happening to their familiar partners (Figure 1A), with six relevant control paradigms (Figures 1B, 9, 11). Additionally, we introduced methods of fine-scale behavioral analysis and state-space behavioral characterization. This allowed us to overcome paradoxical and inconclusive results commonly observed in the traditional analyses of standard behavioral tests when the speculated emotions underlying behaviors are subtle and complex (Ramos, 2008). Finally, we discussed a potential psychological framework that summarizes our observations in mouse stress incubation, which might provide insights on prevention, detection, and treatments of human stress incubation.

## Results

### Long-Term Effects Emerged After Induction by Witnessing an Acute Defeat of a Partner

To minimize the period of stress induction and root out the prolonged development of physical injuries, we developed an assay of witnessing an acute defeat (Figure 1A): Partnership between the male focal mouse and its male partner was established by housing them together for 3 weeks (Day-21–Day 0). To minimize potential hierarchy effects, we selected pair-housed mice without observation of home-cage dyadic agonistic interactions (Horii et al., 2017). During this pair-housing, the mice slept together in a single nest which they built and no aggressive interaction (attacks, pursuits, and over-allogrooming) was observed. On Day 0 (exposure to witnessing stress), the focal mouse observed its partner being attacked by five different aggressor mice in succession (aggressive encounters) and stayed together with the attacked partner between each aggressive encounter (stress infiltration and resting). After the last aggressive encounter, the focal observer mouse [Partner-Observing-Isolated (ParObsIso) mouse] was socially isolated for 4 weeks (Day 0–Day 28). Behavior was tested on Days −7, 1, 7, and 28.

To differentiate behavioral consequences from the exposure to witnessing stress and the effects of isolation, adaptation to the tests, and aging, we first compared ParObsIso mice with a control group of mice isolated from their partners on Day 0 without exposure to witnessing stress [No-Scenario-Isolated (xScenIso) mice; Figure 1B]. We found that ParObsIso mice (*n* = 47) built nests with significantly higher walls than those constructed by xScenIso mice (*n* = 47) after isolation (Figure 9A). ParObsIso mice also increased their body mass in the late phase of the study (Figure 9B).

To further explore potential physiological changes related to witnessing stress underlying the paradigm, we examined corticosterone concentrations in blood plasma. Compared with xScenIso mice, ParObsIso mice showed higher baseline plasma corticosterone level (CORT) after defeat, which reached statistical significance on Day 28 (Figure 9C). In this experiment, we also compared CORT of ParObsIso mice with that of their partners, the attacked mice isolated after defeat, here named Directly attacked-isolated (DirAtcIso) mice (*n* = 5, note that 2 out of 5 mice died on Days 4 and 5, respectively, without obvious physical injury; notably, such losses were not observed in the directly attacked mice which were subsequently group-housed). The tendency of higher CORT was also observed in DirAtcIso mice which had a more obvious CORT increment during the early phase.

**FIGURE 2:**
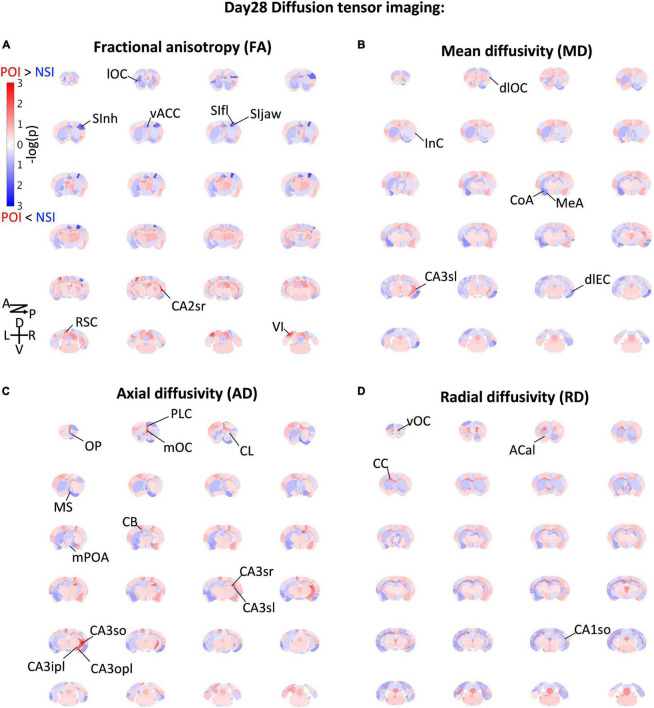
Brain-wide microstructural changes measured by DTI fractional anisotropy **(A)**, mean diffusivity **(B)**, axial diffusivity **(C)**, and radial diffusivity **(D)**. Po, ParObsIso mice; Nsi, xScenIso mice; -log(p), statistical significance through Tukey’s range test; A, anterior; P, posterior; D, dorsal; V, ventral; L, left; R, right; lOC, lateral orbital cortex; SInh, non-homunculus region of the primary sensory cortex; SIfl, forelimb region of the primary sensory cortex; SIjaw, jaw region of the primary sensory cortex; vACC, ventral region of the anterior cingulate cortex; CA2sr, stratum radiatum of the hippocampal cornu ammonis (CA) 2 area; RSC, the retrosplenial cortex; VI, the primary visual cortex; dlOC, dorsolateral orbital cortex; InC, the insular cortex; CoA, the cortical amygdalar nucleus; MeA, medial amygdalar nucleus; CA3sl, stratum lucidum of the hippocampal CA3 area; dlEC, dorsolateral entorhinal cortex; OP, olfactory peduncle; PLC, prelimbic cortex; mOC, medial orbital cortex; CL, claustrum; MS, medial septal complex; mPOA, medial preoptic area; CB, cingulum bundle; CA3sr, stratum radiatum of the hippocampal CA3 area; CA3sl, stratum lucidum of the hippocampal CA3 area;CA3so, stratum oriens of the hippocampal CA3 area; CA3ipl, inner pyramidal layer of the hippocampal CA3 area; CA3opl, outer pyramidal layer of the hippocampal CA3 area; vOC, ventral orbital cortex; ACal, anterior limb of the anterior commissure; CC, corpus callosum; CA1so, stratum oriens of the hippocampal CA1 area.

**FIGURE 3:**
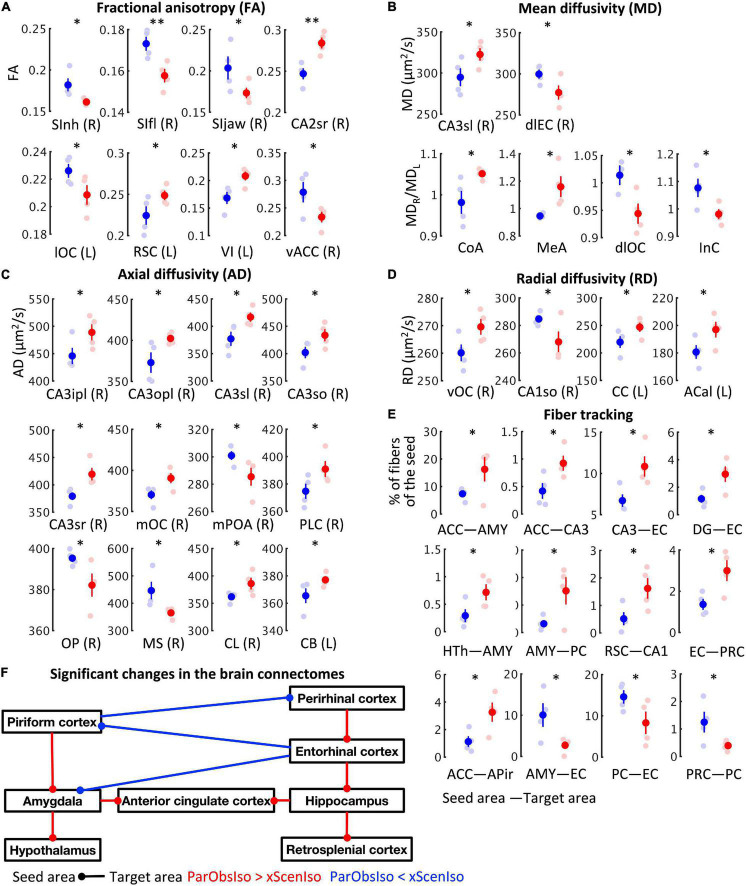
Long-term effects on brain connectivity. **(A)** Fractional anisotropy (FA) of DTI-based water diffusivity suggests the changes of average microstructural integrity in multiple areas of the cerebral cortex. (R), the right area; (L), the left area; *n*_*xScenIso*_ = 4; *n*_*ParObsIso*_ = 4. **(B)** DTI-based mean water diffusivity (MD) suggests the changes of membrane density in multiple areas of the entorhinal cortex-hippocampus system and the straitening of structural hemispheric specializations in the amygdala-insular cortex system. **(C)** DTI-based axial water diffusivity (AD) suggests the changes of neurite organization in multiple areas of the cerebral cortex and white matter mainly in the right hemisphere. **(D)** DTI-based radial water diffusivity (RD) suggests the changes of myelination in multiple areas of the cerebral cortex in the right hemisphere and the white matter in the left hemisphere. **(E)** DTI-based network-wise fiber tracking reveals specific chronic changes of structural connectivity in the brain. **(F)** Trauma-induced structural changes of the underlying brain connectome revealed a network enhancement centered at the anterior cingulate cortex. *, 0.01 ≤ *p* < 0.05; ^**^, 0.001 ≤ *p* < 0.01.

To obtain indication of microstructural changes in the brain, we used diffusion tensor imaging (DTI) of brains collected on Day 28 to analyze brain-wide microstructural differences (Figures 2, 3A–E): Rather than a structural change in the hypothalamus which modulates CORT (DeMorrow, 2018), different measures of DTI-based water diffusivities (Figures 2C–F) were generally higher in the cerebral cortex and hippocampus of ParObsIso than in xScenIso mice. Interestingly, we observed an obvious asymmetry of the brain areas: Gray matters including the cerebral cortex and hippocampus showed differences in the right hemisphere, while white matters including the corpus callosum, anterior commissure, and cingulum bundle showed differences in the left hemisphere. The asymmetry of diffusivity between the right and left hemispheres were most significant in the amygdala and areas of the lateral cerebral cortical subnetwork for ParObsIso mice. For long-range connections, we revealed defeat-induced changes in the perirhinal cortex–entorhinal cortex–hippocampus system, the retrosplenial cortex–hippocampus system, the piriform cortex–amygdala system, and the amygdala–hypothalamus system (Figure 2G), all centered at the anterior cingulate cortex (ACC; Figure 3F).

### Fine-Scale Behavioral Analysis Revealed Gradual Development of Stress Reactions

**FIGURE 4:**
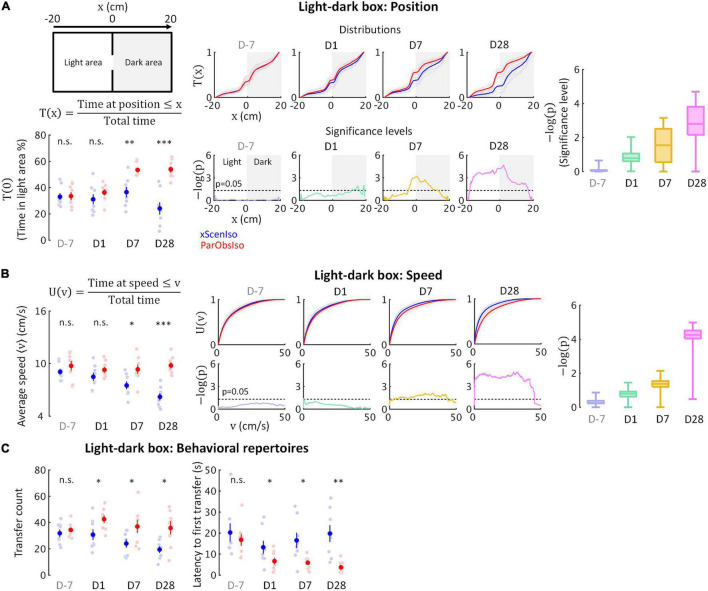
Fine-scale behavioral analysis in light-dark box test detects the gradually developing process of the behavioral difference. **(A)** Light-dark box test quantified through the cumulative position probability T(x) along the light-dark axis (left; total time = 300 s). On average, ParObsIso mice (red) spent more time in the light area than xScenIso mice (blue) during the late post-defeated period [T(0), bottom-left]. Spatially fine-scale behavioral analysis reveals significant differences between ParObsIso and xScenIso populations already in the early post-defeated period (middle). For each position, we compute the mean T(x) across the xScenIso and ParObsIso populations and compute statistical significance through a two-population Student’s *t*-test. These differences gradually increased, as evidenced by significance distributions collapsed across all positions (right; box plots show the minima, lower quartiles, medians, upper quartiles, and maxima). *n*_*xScenIso*_ = 8, *n*_*ParObsIso*_ = 8. **(B)** We similarly quantified speed using the fine-scale cumulative distribution U(v) of having speed ≤v and we show the statistical analysis of population differences in U(v). Cumulative distribution functions of locomotion speed [U(v) of having speed ≤v] and corresponding significance distributions provide an additional independent behavioral index that showed a gradually increasing differences of higher speed in ParObsIso mice. *n*_*xScenIso*_ = 8, *n*_*ParObsIso*_ = 8. **(C)** Higher transfer counts and shorter latency to the first transfers in ParObsIso mice suggest their higher activity and exploratory motivation, respectively (*n*_*xScenIso*_ = 8, *n*_*ParObsIso*_ = 8). *, 0.01 ≤ *p* < 0.05; ^**^, 0.001 ≤ *p* < 0.01; ^***^, *p* < 0.001.

Behavioral tests for rodent anxiety-like reactions were designed to evaluate their stress reaction against their willingness to explore (e.g., light-dark box and elevated plus-maze) or their activity under conditions with a gradient of uncertainty (e.g., open field). We first examined spontaneous behaviors in the light-dark box test (Figure 4; *n* = 8 mice for each group) where the stressor was a natural aversion to brightly lit areas (Kumar et al., 2013). While the time spent in the light area did not differ significantly between xScenIso and ParObsIso mice on Day 1, ParObsIso mice surprisingly spent more time in the light area than xScenIso mice on Days 7 and 28 (Figure 4A, left panels; *p*_*D7*_ = 0.003 and *p*_*D28*_ < 0.001, Tukey’s range test). This result raised the following questions: (i) Did this behavioral difference start to develop immediately after the defeated event or only after a delay? (ii) What comparative emotionality does this behavioral difference indicate?

To answer (i), we examined the positions of the mice in the light-dark box on a fine-scale. Based on spatial symmetry, we analyzed T(x), the cumulative probability distribution of time that the mouse spent at positions along the axis of the light-dark box (Figure 3A, top-right scheme and the equation, and middle panels for the results). We calculated significance levels [presented as -log of *p*-values, -log(p)] of T(x) between ParObsIso and xScenIso populations by computing position-dependent population means and applying a two-tailed, two-sample Student’s *t*-test (Figure 4A, bottom-middle panels). Already on Day1, ParObsIso mice showed differences in their spatial distribution, as they spent less time than xScenIso mice at the far end of the dark area. This tendency increased with time: On Day 7, ParObsIso mice spent more time in the light area close to the door compared to xScenIso, and then on Day 28 additionally on the far side of the light area. Collapsed -log(p) distributions reveal the overall gradual increase in spatial preference differences (Figure 4A, right panel). Additionally, ParObsIso mice maintained a higher locomotor speed compared to the gradually decreasing speed of xScenIso mice (Figure 4B) and showed more transfers between the boxes (Figure 3C, left panel) and shorter latencies until their first transfers from Day 1 (Figure 3C, right panel).

**FIGURE 5:**
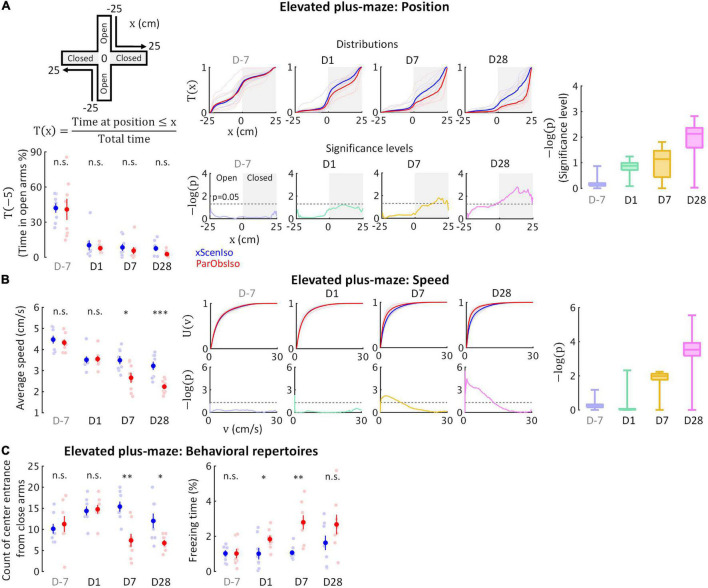
Behavioral testing in the elevated plus-maze demonstrates that stress incubation of anxiety caused the observed differences. **(A)** ParObsIso and xScenIso mice did not differ significantly in the time they spent in opened arms (total time = 300 s); however, spatial distributions show differences in preferred location between ParObsIso and xScenIso mice in the closed arms, which increased with time (*n*_*xScenIso*_ = 8, *n*_*ParObsIso*_ = 8). **(B)** Cumulative distribution functions of locomotion speed and corresponding significance distributions show a gradually increasing differences of lower speeds in ParObsIso mice (*n*_*xScenIso*_ = 8, *n*_*ParObsIso*_ = 8). **(C)** Less exploration from close arms to platform center and longer freezing time in ParObsIso mice suggest their stronger stress reactions (*n*_*xScenIso*_ = 8, *n*_*ParObsIso*_ = 8). *, 0.01 ≤ *p* < 0.05; ^**^, 0.001 ≤ *p* < 0.01; ^***^, *p* < 0.001.

Regarding (ii), we additionally examined behaviors of xScenIso and ParObsIso mice in the elevated plus-maze test (Figure 5A; *n* = 8 mice for each group) where stressors included fear of falling and exposure. After first exposure on Day-7 and separation, mice spent only a fraction of the time in the open arms of the maze, but with no significant difference between xScenIso and ParObsIso mice (Figure 5A, left panels). However, ParObsIso mice spent increasingly more time in the far end of the closed arms (Figure 5A, middle panels) and moved more slowly in the elevated plus-maze after stress (Figure 5B) with longer periods of freezing (Figure 5C, right panel) and fewer entries to the central platform from the closed arms (Figure 5C, left panel). Although the gradually increasing differences between xScenIso and ParObsIso mice (Figures 5A,B) was consistent, the opposite tendency of reactions in the light-dark box and the elevated plus-maze tests was seemingly paradoxical.

Reactions of cognitive anxiety are expected to be opposite of those shown in somatic anxiety (Cloninger, 1988). We compared the behaviors of xScenIso and ParObsIso mice in the tests with the tested behaviors of mice injected with caffeine which induces anxiety somatically (DSM-5, 2013), and mice after experiencing brief shocks which induces anxiety cognitively (Bolton and Robinson, 2017), under the otherwise same experimental conditions and procedures. Consistent with the observations reported in the literature (Suarez and Gallup, 1981; Borsini et al., 2002; Gulick and Gould, 2009; Wu et al., 2017), the behavioral characteristics of caffeine-injected mice and foot-shocked mice in standard tests are paradoxical when applying traditional analyses [Supplementary Figures 1A,B (left panel)]. We first evaluated the local likelihood of a given behavioral state described by locomotion matrices to be recorded from a caffeine-injected, foot-shocked, or non-treated mouse (Supplementary Videos 1–6), and then calculated the global likelihood of behaviors of xScenIso and ParObsIso mice in a test to be caffeine-injected-like, foot-shocked-like, or non-treated-like. While xScenIso mice kept showing non-treated-like behaviors in both tests although their behaviors in classical analyses changed, ParObsIso mice developed caffeine-injected-like behaviors in the light-dark box test and developed foot-shocked-like behaviors in the elevated plus-maze test after stress (Supplementary Figures 1C,D).

### Different Developmental Components Untangled From a Single Behavioral Test

**FIGURE 6:**
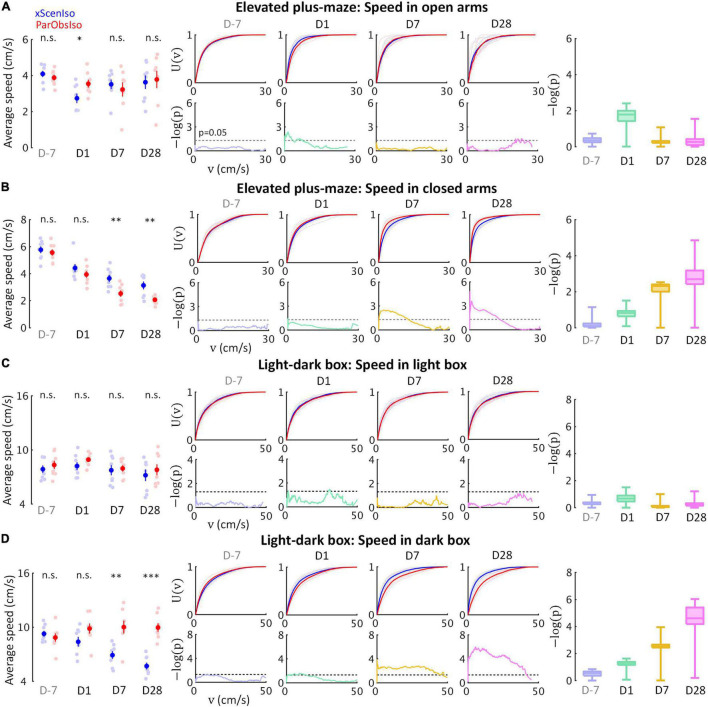
Gradually increasing anxiety and acute fear reaction were untangled in standard behavioral tests as different psychological components with distinctive developments. Locomotion speed shows acute difference only in stressor zones [open arms **(A)** and light area **(C)**], but incubated differences in stressor-free zones [dark area **(B)** and closed arms **(D)**] (*n*_*xScenIso*_ = 8, *n*_*ParObsIso*_ = 8). *, 0.01 ≤ *p* < 0.05; ^**^, 0.001 ≤ *p* < 0.01; ^***^, *p* < 0.001.

We further observed that, in the light-dark box and elevated plus-maze tests, key incubation features were consistently more obvious within stressor-free zones (dark area and closed arms) than within stressor zones (light area and open arms). We analyzed locomotor speed, as an independent behavioral index of mouse position, separately in stressor-free and stressor zones (Figure 6). As expected, two different behavioral patterns developed in the two zones: While speed differences consistently increased in stressor-free zones (Figures 6B,D), speed differences in stressor zones only showed acute increases on Day 1 (Figures 6A,C).

**FIGURE 7:**
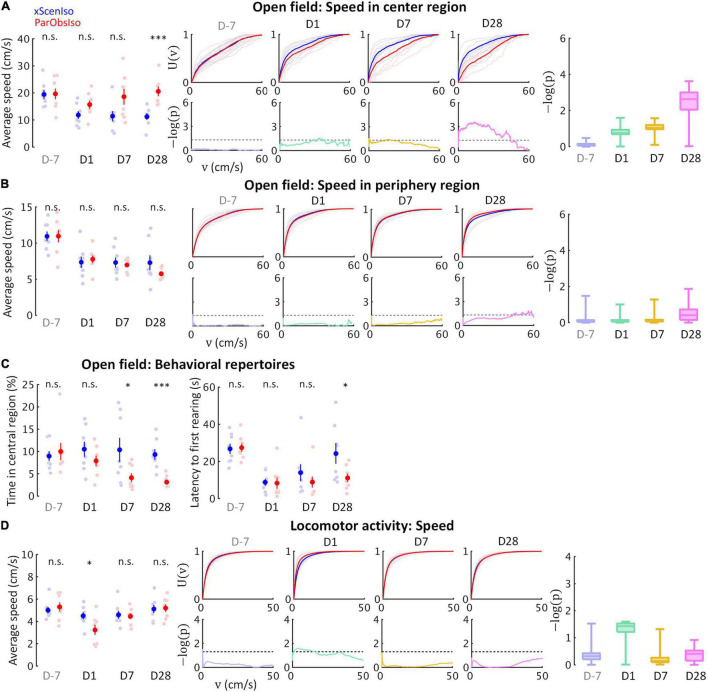
Behavioral testing in the open field test and locomotor activity test confirms distinctive psychological substrates and their corresponding development patterns. **(A)** Anxiety is evident in the open field test through a delayed onset of locomotor speed differences in the center region with higher spatial uncertainty (*n*_*xScenIso*_ = 8, *n*_*ParObsIso*_ = 8). **(B)** The differences of locomotor speed observed in the center region did not occur in the periphery region with lower spatial uncertainty (*n*_*xScenIso*_ = 8, *n*_*ParObsIso*_ = 8). **(C)** Less time spent in the central region by ParObsIso mice suggests their avoidance of a region with high special uncertainty, while shorter latency to their first rearing indicates their higher exploratory motivation (*n*_*xScenIso*_ = 8, *n*_*ParObsIso*_ = 8). **(D)** In the locomotor activity test without stressors, acute effects of activity reduction recovered in the later post-defeated period (*n*_*xScenIso*_ = 8, *n*_*ParObsIso*_ = 8). *, 0.01 ≤ *p* < 0.05; ^***^, *p* < 0.001.

Fear is a response to a known threat with a magnitude that increases with the strength of the threat, whereas anxiety is a response to uncertainty with a magnitude that increases with the uncertainty of a situation (Grupe and Nitschke, 2013). To better understand the two developmental patterns, we examined mouse locomotor speed in relatively uncertain and secure environments separately, using the open field test (Figures 7A–C; *n* = 8 mice for each group) and the locomotor activity test (Figure 7D; *n* = 8 mice for each group), respectively. Compared to xScenIso mice, ParObsIso mice moved faster in the center region of the open field (Figure 7A), which was not observed in the periphery (Figure 7B). The difference between groups increased gradually toward the center but not in the periphery (Figures 7A,B). The avoidance of spatial uncertainty by ParObsIso mice was also reflected in the shorter time they spent near the center (Figure 7C, left panel) and showed a shorter latency to the first rearing during the test (Figure 7C, right panel). In contrast, ParObsIso mice moved significantly slower on Day 1 in the locomotor activity test and recovered on Days 7 and 28 (Figure 7D).

### Social Differences Weakly Depend on Emotional Differences and Vice Versa

**FIGURE 8:**
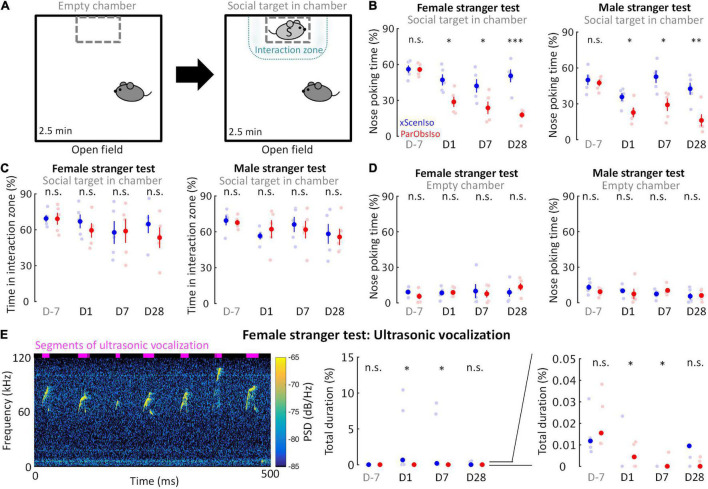
Acute psychosocial defeat decreases social interest. **(A)** The active social interaction test with consecutive non-social and social phases. During the social phase, motivation for social contact toward a stranger mouse (light gray S) was evaluated as the time spent for social approaches of nose poking and the time spent in the delineated interaction zone. **(B)** ParObsIso mice made fewer nose poking to both female (left) and male (right) strangers (*n*_*xScenIso*_ = 5, *n*_*ParObsIso*_ = 5). **(C)** There was no significant difference in the time spent in the interaction zone during the social phase, suggesting a decrease of social interest instead of an active social avoidance (*n*_*xScenIso*_ = 5, *n*_*ParObsIso*_ = 5). **(D)** There was no significant difference in the time spent of nose poking during the non-social phase, confirming that the observed differences of nose poking time stemmed from a specifically social root (*n*_*xScenIso*_ = 5, *n*_*ParObsIso*_ = 5). **(E)** Spectrogram of short but conspicuous ultrasonic vocalizations emphasizes a specific behavioral repertoire during the social session in the female stranger test of a xScenIso mouse on Day 1. More vocalization was recorded from xScenIso mice than ParObsIso mice on Days 1 and 7. Reduced ultrasonic vocalization during the social session of the female stranger test in ParObsIso mice attests to diminished social communication (*n*_*xScenIso*_ = 5, *n*_*ParObsIso*_ = 5). Note that data points greater than 0.05% are not visible in the right panel which zoom in the data of the middle panel to emphasize the data distributions in the range of 0–0.05%. Data points and median, one-tailed Mann–Whitney *U*-test; PSD, power spectral density. *, 0.01 ≤ *p* < 0.05; ^**^, 0.001 ≤ *p* < 0.01; ^***^, *p* < 0.001.

Emotional responses simplify and speed up animal reactions to complex external cues and are critical in corresponding social interactions (Anderson, 2016). We examined mouse social motivation in a two-session social test (Figure 8A; *n* = 5 mice for each group) where a non-social session was followed by a social session. During the social session, ParObsIso mice spent less time in social approaches of nose poking toward both female and male strangers, starting from Day 1, and remained less social compared to xScenIso mice during the post-defeated period (Figure 8B and Supplementary Figure 2). The time they spent in the interaction zone around the social target, however, did not differ significantly from that of xScenIso mice (Figure 8C). Both, ParObsIso and xScenIso mice, spent only a short but similar time on nose poking during the non-social session through the recordings on different days, with no significant difference between ParObsIso and xScenIso populations (Figure 8D), indicating that the observed difference of nose poking time was specific to social behavior. In addition, less social vocalization was recorded during the female stranger test for ParObsIso mice (Figure 8E).

**FIGURE 9:**
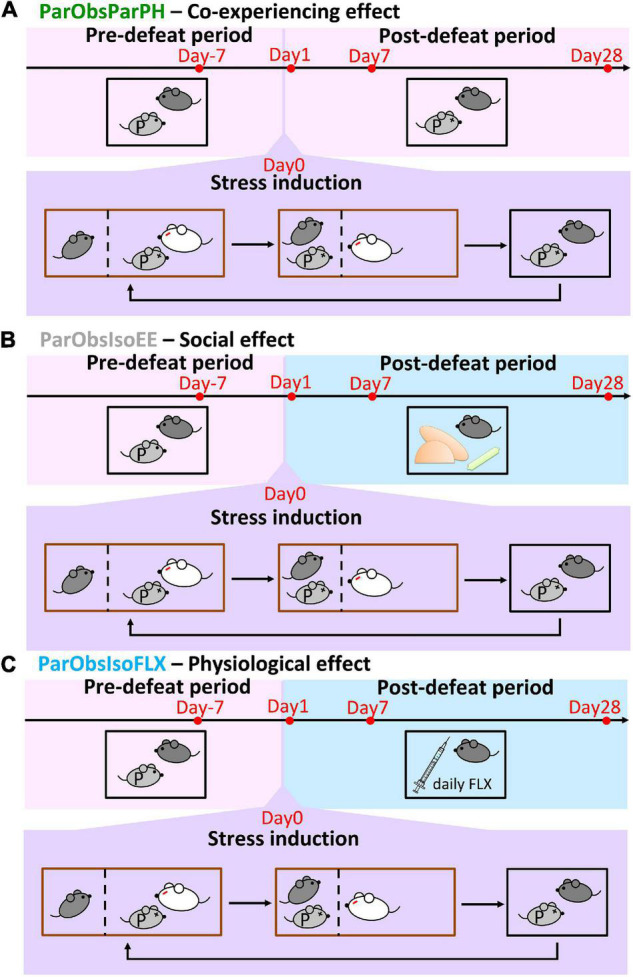
Control paradigms with different post-defeat environments. **(A)** Partner-Observing-Partner-Pair-Housed (ParObsParPH) mice were pair-housed with their partners after exposure to witnessing stress and identified the social transfer effect of co-experiencing defeat in the behavioral paradigm. **(B)** Partner-Observing-Isolated-Environment-Enriched (ParObsIsoEE) mice were provided with toys after exposure to witnessing stress and identified the social rescue effect in the behavioral paradigm. **(C)** Partner-Observing-Isolated-Fluoxetine-Treated (ParObsIsoFLX) mice were treated with fluoxetine after exposure to witnessing stress and identified the pharmacological rescue effect in the behavioral paradigm.

**FIGURE 10:**
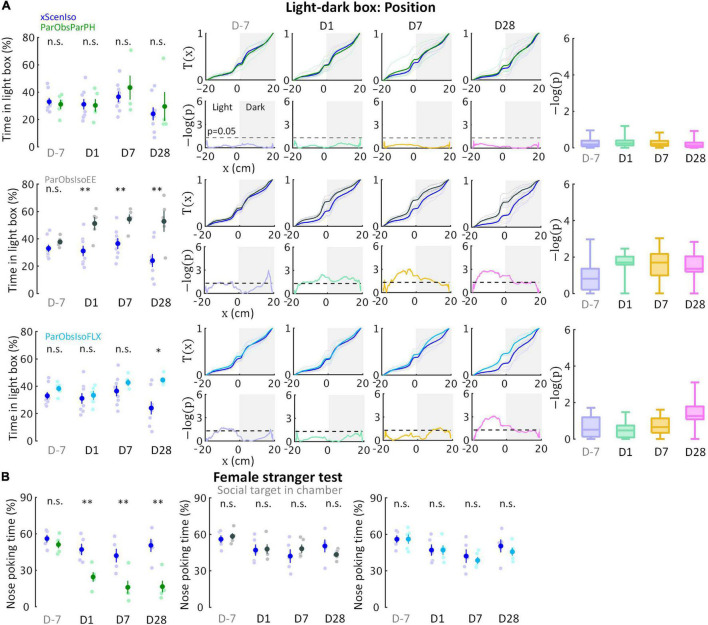
Developments of emotional and social differences are not inter-dependent. **(A)** Positions in the light-dark box test indicate that ParObsParPH mice did not develop the chronic stress reactions, ParObsIsoEE mice developed chronic stress reactions which were stronger than that of ParObsIso mice in the early phase, and ParObsIsoFLX developed stress reactions in the late phase (*n*_*xScenIso*_ = 8, *n*_*ParObsParPH*_ = 5, *n*_*ParObsIsoEE*_ = 5, *n*_*ParObsIsoFLX*_ = 5). **(B)** Nose poking times in the female stranger test indicate that ParObsParPH, but not ParObsIsoEE and ParObsIsoFLX, mice develop the social differences of ParObsIso mice (*n*_*xScenIso*_ = 5, *n*_*ParObsParPH*_ = 5, *n*_*ParObsIsoEE*_ = 5, *n*_*ParObsIsoFLX*_ = 5). *, 0.01 ≤ *p* < 0.05; ^**^, 0.001 ≤ *p* < 0.01.

An important condition in our behavioral paradigm was the forced social isolation after defeat. We examined the impact of post-defeated social condition on developments by the second control group of mice [Partner-Observing-Partner-Pair-Housed (ParObsParPH) mice; Figure 9A (*n* = 5 mice for each experiment)] as each of them was kept pair-housed with its attacked partner after exposure to witnessing stress. Noteworthily, developments of the differences in spontaneous behaviors did not occur in ParObsParPH mice (Figure 10A, upper panels and Supplementary Figure 3), but importantly, developments of behavioral differences in social interactions showed the differences of ParObsIso mice (Figure 10B, left panel).

To test the potential significance of the post-defeated social factor in developments, we conducted two additional control experiments with the third control group of mice where each of them was provided with toys during social isolation after exposure to witnessing stress [Partner-Observing-Isolated-Environment-Enriched (ParObsIsoEE) mice; Figure 9B (*n* = 5 mice for each experiment)] and the fourth control group [Partner-Observing-Isolated-Fluoxetine-Treated (ParObsIsoFLX) mice; Figure 9C (*n* = 5 mice for each experiment)] as each of them was injected with fluoxetine daily after defeat. ParObsIsoEE mice showed similar nose poking times in the female stranger test as xScenIso mice (Figure 10B, middle panel); however, they showed the behavioral differences of ParObsIso mice in the light-dark box test, which even had a stronger difference starting from the early phase (Figure 10A, middle panels). For ParObsIsoFLX mice, while they showed less behavioral difference during the early post-defeated phase in the light-dark box test (Figure 10A, bottom panels) and did not display a reduction of nose poking times in the female stranger test (Figure 10B, right panel), their behavioral difference in the light-dark box test reached significance in the late phase and the behavioral difference in elevated plus-maze test was more obvious in the closed arms after exposure to witnessing stress (Figure 10A, bottom panels).

### Social Relationship Determined Context-Wide Development Over Stress Incubation

**FIGURE 11:**
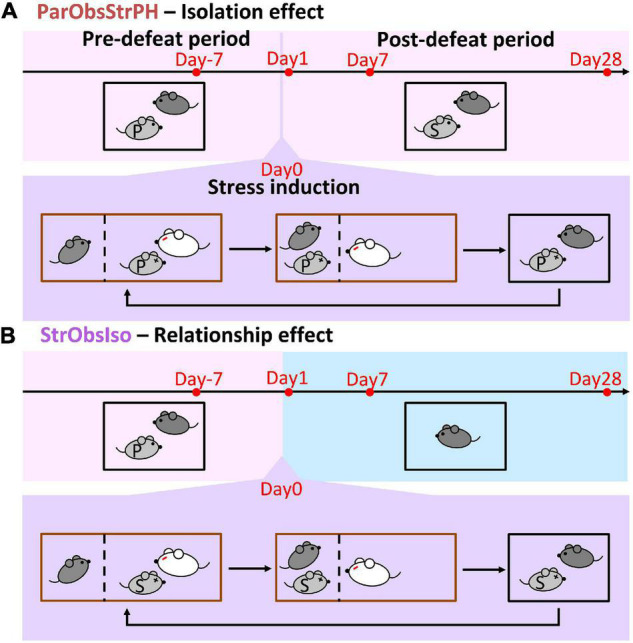
Control paradigms with conditions of stranger mice. **(A)** Partner-Observing-Stranger-Pair-Housed (ParObsStrPH) mice were pair-housed with strangers after exposure to witnessing stress and identified the isolation effect in the behavioral paradigm. **(B)** Stranger Observing Isolated (StrObsIso) mice had witnessing experience of defeat that happened to strangers, rather than to their pair-housed partners, and identified the relationship effect in the behavioral paradigm.

**FIGURE 12:**
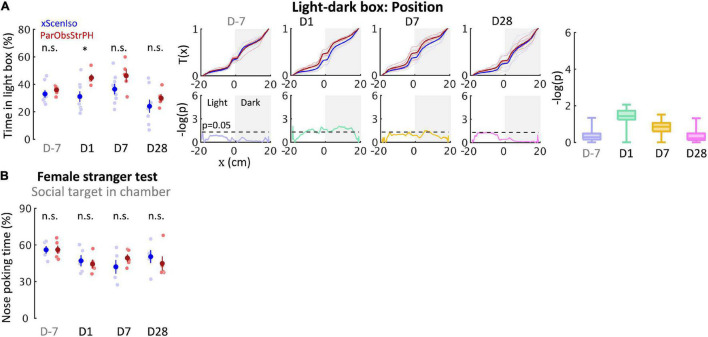
Control experiments identify necessity of relationship-dependent vicarious defeat for anxiety incubation. **(A)** Positions in the light-dark box test indicate that ParObsStrPH mice did not develop the behavioral differences in the late phase, although ParObsStrPH mice displayed the difference in the early phase (*n*_*xScenIso*_ = 8, *n*_*ParObsStrPH*_ = 5, *n*_*xAggrExpIso*_ = 5). **(B)** Nose poking times in the female stranger test indicate that ParObsStrPH mice did not develop the social differences (*n*_*xScenIso*_ = 5, *n*_*ParObsStrPH*_ = 5, *n*_*xAggrExpIso*_ = 5). *, 0.01 ≤ *p* < 0.05.

To further address social factors for the developments of behavioral differences, we conducted the fifth control experiment where each of the mice was kept pair-housed with a stranger after exposure to witnessing stress [Partner-Observing-Stranger-Pair-Housed (ParObsStrPH) mice; Figure 11A (*n* = 5 mice for each experiment)], altering the post-defeated social relationship in the ParObsParPH paradigm. In the ParObsStrPH paradigm, if the pair-housed strangers were the socially defeated intruders, we observed aggressive attacks or inter-male mounting toward strangers by all focal mice (*n* = 5 out of 5) during their pair-housing after defeat. Similar aggression was observed among ParObsIso mice, but not toward their defeated partners, when they were group-housed after exposure to witnessing stress. Because of these observations of partnership-dependent aggressive or non-aggressive behaviors, we focused on behavioral tests of the ParObsStrPH mice pair-housed with non-defeated strangers and recorded their behavior. ParObsStrPH mice did not display the development of behavioral differences in the light-dark box, elevated plus-maze, and female stranger tests (Figure 12A, upper panels; Figure 12B, left panel and Supplementary Figure 3). Only their position in the light-dark box test showed an acute difference (Figure 12A, upper panels).

**FIGURE 13:**
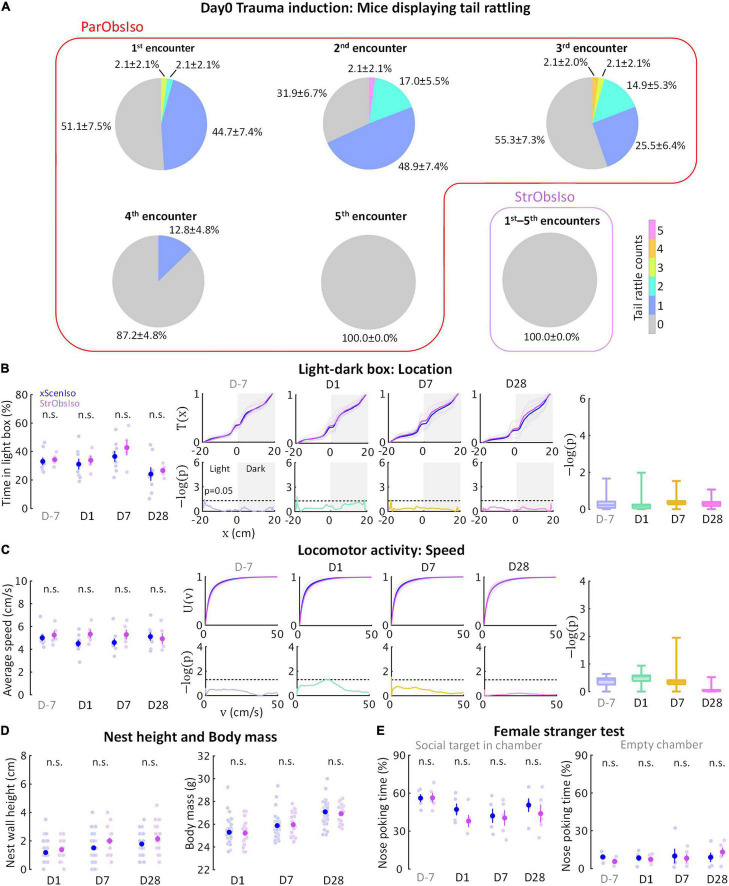
Social relationship in exposure to witnessing stress determines stress development. **(A)** Social relationship increased emotional impact, evidenced by tail rattling behavior during exposure to witnessing stress (*n*_*xScenIso*_ = 47, *n*_*StrObsIso*_ = 20). **(B–E)** No significant acute or chronic difference was found in StrObsIso mice (B and C, *n*_*xScenIso*_ = 47, *n*_*StrObsIso*_ = 20; D, *n*_*xScenIso*_ = 8, *n*_*StrObsIso*_ = 5; E, *n*_*xScenIso*_ = 5, *n*_*StrObsIso*_ = 5).

Following the evidence that social relationship governs the stress development, we further examined the sixth group of mice [Stranger-Observing-Isolated (StrObsIso) mice; Figure 11B (*n* = 5 mice for each experiment)], each of the mice observed different stranger mice being attacked by different aggressors and stayed together with each stranger between aggressive encounters before isolation. During stress induction, two notable behavioral differences were observed: While tail rattling during aggressive encounters and hiding under bedding material with the partner during resting were observed in 83% (*n* = 39 out of 47 mice) and 100% (*n* = 47 out of 47 mice) ParObsIso mice (Supplementary Video 8), respectively, no such behaviors were shown by StrObsIso mice (*n* = 0 out of 20 mice; *n* = 0 out of 20 mice). In ParObsIso mice, the frequency of tail rattles dropped progressively during aggressive encounters (Figure 13A), representing a transient reaction during exposure to witnessing stress. Moreover, long-lasting behavioral differences were not observed in StrObsIso mice (Figures 13B–E), which further excluded potential effects from salient, non-specific environmental manipulation (e.g., rotation through aggressors’ home cages) and sensory shock (e.g., olfactory cues from urine and vocalization indicating fear) during exposure to witnessing stress. Taken together, these results show that social relationship constitutes as a critical factor of both exposure to witnessing stress and its following context-wide developments of behavioral differences.

**FIGURE 14:**
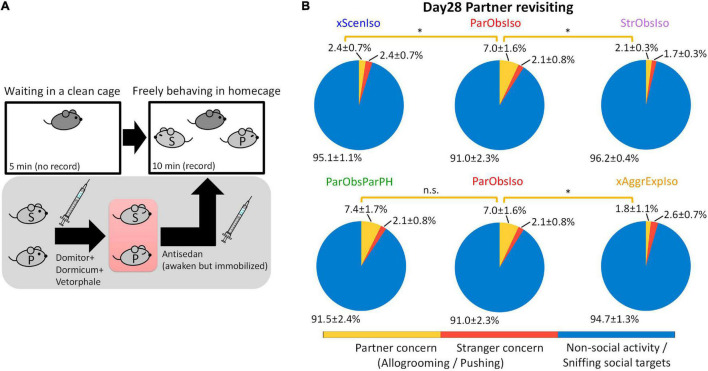
Long-term memory of partnership correlates with anxiety incubation. **(A)** The partner-revisiting test. A stranger mouse (light gray S) and the previously pair-housed partner (light gray P), both immobilized, were presented as social targets. Pink rectangle, heating pad. **(B)** ParObsIso and ParObsParPH mice showed significantly longer allogrooming or pushing their partners (yellow, % of time spent in partner concern behavior) than either xScenIso, xAggrExpIso, or StrObsIso mice (*n*_*xScenIso*_ = 5, *n*_*ParObsIso*_ = 5, *n*_*StrObsIso*_ = 5, *n*_*ParObsParPH*_ = 5, *n*_*xAggrExpIso*_ = 5). Standard errors were calculated from bootstrapped data. *, 0.01 ≤ *p* < 0.05.

We examined the persistence of social memory (Kogan et al., 2000; Okuyama et al., 2016) in a partner-revisiting test on Day 28 (Figure 14A; *n* = 5 mice for each group). Social stimuli were the previous partner and a stranger mouse, both immobilized to allow enough social cues to be attractive, but no active interaction with the focal mouse. To avoid possible influences of social cues from socially defeated mice, stranger mice used to test xScenIso and StrObsIso mice were partners of ParObsIso mice. Strikingly, ParObsIso mice as well as ParObsIsoPH mice, which were separated from their partners right before their partners got immobilized for the tests, spent three times as much time allogrooming or pushing their previous partners as did xScenIso, xAggrExpIso, and StrObsIso mice (Figure 14B and Supplementary Video 9).

## Discussion

We present an approach to address otherwise challenging and inconclusive behavioral data and use it to study stress incubation in laboratory mice. The results demonstrate a system-level view of experimentally disentangled components, processes, and determinants in stress incubation. The fine-scale behavioral analyses we introduced here provides a simple, non-invasive analytic tool to capture informative behavioral details and is not limited to laboratory animals. We also report the asymmetry of brain-wide microstructural changes and the strengthening of an ACC-centered network in mice after acute witnessing social stress. Based on the context-wide observations from our experiments, we demonstrate a significant link between social relationship and stress incubation in mice. Our study provides both technical and conceptual advances which could be considered in the study of human psychiatry disorders.

### Detection and Identification of Animal Emotion

The traditional approach to identify animal emotion is to test if animals show particular behaviors specified by the experimental test assumed when it was designed (Walf and Frye, 2007). Although a few studies have considered human-unique, animal-unique, and human-animal-sharing emotions (Anderson and Adolphs, 2014), animal studies frequently use multiple tests to assess the same psychological factor and report the results that are consistent with the expectation to emphasize its face validity, predictive validity, and construct validity from human analogies (Calhoon and Tye, 2015). However, experimental animals normally display obvious but not inter-supporting behaviors in different tests with the same logic and assumptions (Ramos, 2008). Furthermore, even if the behavioral results are consistent with the expectation, they can still be alternatively explained (Garcia et al., 2008). The limitations can be due to circular arguments embedded in a reductive logic. This ambiguity reaches deeply into the history of widely used behavioral tests and therefore have resulted in a considerable amount of inconclusive and seemingly paradoxical results, which are usually left for discussion or remain unreported (Hascoët et al., 2001; Carobrez and Bertoglio, 2005; Engin and Treit, 2008; Crusio, 2013; Ennaceur, 2014; Kulesskaya and Voikar, 2014; Henriques-Alves and Queiroz, 2016).

Discussing potential interpretations or possible factors of these massive observations can be endless and easily lead to skeptical arguments especially if the psychobehavioral substrate is complex. Founded on the development of computational ethology (Chen et al., 2013; Anderson and Perona, 2014; Forkosh et al., 2019; Nath et al., 2019; Sangiamo et al., 2020; Sturman et al., 2020), estimating and testing a consistent overview of complex observations can be more reliable. In this study, we introduced fine-scale behavioral analysis and state-space behavioral characterization to access animal emotion from standard behavioral tests which give inconclusive results when analyzed in the traditional way. We were first able to discover subtle behavioral differences and recognize otherwise obscured behavioral details in stress incubation with the richer measurements. With these data representations, rather than based on the expectation of presumed behaviors as traditionally done, we can further address psychological meaning by quantifying how high-dimensional behaviors correlated with given physical variables in the testing environments. Our examples using computational ethology approaches provide new insights beyond traditional interpretations of animal performances in standard behavioral tests.

### From Stress Incubation in Mice to Human Affective Disorders

Stress incubation has long been recognized clinically in human, occurring in social anxiety disorder, depressive disorder, bipolar disorder, binge-eating disorder, and PTSD (Bebbington et al., 1993; Malkoff-Schwartz et al., 2000; Wainwright and Surtees, 2002; Pike et al., 2006; Dunn et al., 2019). Although we focused on stress incubation in mice, our work may provide several important insights to complex human psychiatry. Behavioral paradigms of laboratory rodents that showed stress incubation have mostly been found in studies aimed to simulate human PTSD (Balogh et al., 2002; Philbert et al., 2011; Warren et al., 2013; Sial et al., 2016). The diversity of these rodent models is even highlighted by a paradigm of stressor-free, pharmacologically induced memory impairments in mice, which was recognized as a model of PTSD and further identified the corresponding pathophysiological mechanism (Kaouane et al., 2012).

In this study, we observed continuous growth of the differences in uncertainty-related spontaneous behaviors while that of uncertainty-unrelated spontaneous behaviors had long vanished (Figures 4–7). The time course of substantial social differences also led the substantial differences of uncertainty-related spontaneous behaviors (Figures 4, 5, 8). Furthermore, pair-housing with partner mice selectively rescued the increased differences of spontaneous behaviors but not social differences (Figure 10), and in contrast, environmental enhancement selectively strengthen the differences of spontaneous behaviors but reduced the differences in social behavior (Figure 10A, middle panels). These observations imply that multiple important features of stress reactions (e.g., the uncertainty-related, uncertainty-unrelated, and social behaviors) may be less interdependent during stress incubation as previously thought.

Even though, we found that the alternation of a single social cognitive factor, social relationship, eliminated this complexity of behavioral differences from the defeated experience (Figures 13, 14). Conceptualization of social support as a “stress buffer” has been proposed to explain the positive association between responsive social resources in a small social network and the adverse effects of stressful events (Cohen and Wills, 1985). Indeed, among all rescue controls, including social, environmental, or pharmacological approaches (Figure 9), pair-housing with non-defeated stranger mice showed the best rescuing effects on diverse developments in our paradigm (Figure 10). Interestingly, a stranger rather than a partner has a stronger effect on preventing behavioral differences in the female stranger test and light-dark box test (Figures 10, 12).

Social relationship regulates the strength of emotional contagion in rodent (Pisansky et al., 2017). We further reported that observer mice showed distinct social reactions to a defeated stranger and to a defeated partner after witnessing the aggressive encounter happened to these directly attacked mice (Figures 9A, 11B). Human studies considered the possibility that simultaneous stress buffering and stress exacerbation in the stress process is associated with social support and social undermining from a closed relationship (Cranford, 2004). Together, these results suggest that how efficient a subject can provide stress buffering effect on another subject is determined by the social cognitive mechanisms of both individuals. While much remains to be considered before drawing conclusions from animal studies and applying them to human disorders, we speculate that stress incubation could be a process governed by a common, cognitive factor, and that this factor underlies the otherwise rather independent developments during stress incubation.

### Anterior Cingulate Cortex-Centered Hemisphere-Specific Correlates of Stress Incubation

According to previous experiments in rodents, a delay in generalized avoidance was proposed to develop from an amplification of fear expression (Houston et al., 1999; Pamplona et al., 2011; Sillivan et al., 2017). Following this line, by correlating with the freezing behavior following a single scrambled foot shock in mice, early inhibition of PTH2R (parathyroid hormone two receptor)-mediated TIP39 (tuberoinfundibular peptide of 39 residues) signaling in the medial amygdalar nucleus was demonstrated to enhance fear memory much later (Tsuda et al., 2015). We extend this view by reporting a more plentiful, varied content of stress development after an acute aversive event from the experimental control groups.

In this context, since the brain heavily integrates not only external but also internal causes (Larkum, 2013; Lee et al., 2014; Donaldson et al., 2015; Lee R.X. et al., 2015; Funamizu et al., 2016; Tononi et al., 2016; Matias et al., 2017; Murugan et al., 2017; Remedios et al., 2017; Kohl et al., 2018; Roome and Kuhn, 2018; Zelikowsky et al., 2018), the potentially slow and global change of brain dynamics may arise from an altered dynamic of particular circuits through their interconnected nodes (Ko, 2017). Indeed, from DTI scanning, we found an enhanced ACC-centered network in the right brain hemisphere 28 days after the witnessing of social stress. This is in agreement with previous finding that the right but not the left ACC controls observational fear learning in mice (Kim et al., 2012). The medial prefrontal cortex (mPFC), which includes ACC and prelimbic cortex in mice, have been reported to exhibit both functional and physiological asymmetry between hemispheres. For examples, the right mPFC was reported to control the acquisition of stress during hazardous experiences while the left mPFC was found to play a dominant role in translating stress into social behavior (Lee E. et al., 2015). Stress-induced mesocortical dopamine activation was also found for the right mPFC but not the left (Sullivan and Gratton, 1998). In human research of trauma-induced stress, the amygdala, mPFC, and hippocampus are the brain regions traditionally focused on (Shin et al., 2006), with morphological differences reported in the right hippocampus (Gilbertson et al., 2002; Pavić et al., 2007).

Additionally, there are interesting findings worth to be jointly discussed. First, we found that the connections between the piriform and perirhinal cortices decreased (Figures 3E,F). The piriform and perirhinal cortices are the two core parahippocampal structures involve in the kindling phenomenon, the daily progressive increase in response severity of both electrographic and behavioral seizure activity (McIntyre, 2006; McIntyre and Kelly, 2006). Although barely discussed, a supposed link between the kindling phenomenon and fear conditioning has been suggested along with rat models of the PTSD (Rau et al., 2005; Knox et al., 2012). Second, we found a right-hemisphere-specific differences of sensory cortical areas (Figures 3E,F). This is consistent with the finding that responses in barrel cortex to social touch differed from responses to conventional tactile stimuli (Bobrov et al., 2014). It is also in line with the lateralization of oxytocin receptor expression found in the auditory cortex of adult female mice (Marlin and Froemke, 2017), which enables maternal behavior (Marlin et al., 2015). Third, we found an asymmetric tendency of microstructure changes in the amygdala. The amygdala has been known for its highly lateralized morphology in adult mice (Pfau et al., 2016) and rats (Johnson et al., 2008). In human, damage to the right but not left amygdala impairs the cortical processing of vocal emotions (Frühholz et al., 2015). These three findings together with the right-hemisphere-specific enhancement of an ACC-centered inter-regional connectivity will guide future attempts to systematically test potential causal roles of top-down circuitry regulation from the prefrontal cortex in disease development.

## Materials and Methods

All animal experiments were approved by the Institutional Animal Care and Use Committee (IACUC). All animal procedures were conducted in accordance with guidelines of the OIST IACUC in an Association for Assessment and Accreditation of Laboratory Animal Care (AAALAC)-accredited facility.

### Study Design

The goal of this work is to identify diverse psychological aspects, temporal patterns, and associations of development in mice after a single exposure to witnessing stress. Pre-specified hypotheses stated that (i) development of behavioral differences is already represented in behavioral details during the early post-defeated phase, while (ii) the behavioral differences of spontaneous behaviors and social interactions are inter-dependent. Our data support the first pre-specified hypothesis, but not the second. All other hypotheses were suggested after initiation of the data analyses.

We developed a novel mouse model of psychosocial defeat under highly controlled conditions (psychosocial manipulations, subjective experiences, and genetic background) and by applying fine-scale analysis to standard behavioral tests. To induce acute witnessing defeat, a pair-housed mouse observed how its partner got bullied by a larger, aggressive mouse on the day of exposure to witnessing stress. After this defeat, the observer mouse was isolated and developed behavioral differences compared to control mice in the ensuing weeks. The control groups included (i) mice isolated without experiencing exposure to witnessing stress, (ii) mice isolated after observing how a stranger mouse got bullied by a larger, aggressive mouse, (iii) mice which were pair-housed with their defeat partners after observing how its partner got bullied by a larger, aggressive mouse, (iv) mice which were pair-housed with strangers after observing how its partner got bullied by a larger, aggressive mouse, (v) mice isolated with their environment enriched, and (vi) mice isolated with daily injections of fluoxetine. The behavioral tests included the light-dark box test, elevated plus-maze test, open field test, locomotor activity test, active social contact test to a female stranger, active social contact test to a male stranger, and partner-revisiting test. The non-behavioral tests included the body mass measurement, nest wall height measurement, baseline corticosterone concentration test, and *ex vivo* DTI.

No statistical methods were used to predetermine sample sizes. Animal numbers were determined based on previous studies (Takahashi et al., 2015). 8-week-old male C57BL/6J mice, 16-week-old female C57BL/6J mice, and 20-week-old or older male Slc:ICR mice were used. No data were excluded. No outliers were defined. Mice were from different litters. Mice were randomly paired. A focal mouse was randomly selected from each pair of mice. Mice were randomly allocated into experimental groups. Testing order among groups was counterbalanced. Strangers and aggressors were randomly assigned. All behavioral tests were conducted in quintuplicate to octuplicate sampling replicates. All behavioral tests were conducted in single to quadrupole experimental cohorts with respect to start dates of a test. All other records were conducted in quadruplicate to septuplicate experimental cohorts.

The endpoints were prospectively selected. Partner mice were expected to get minor injuries from aggressor mice during aggressive encounters; typically, attack bites on the dorsal side of posterior trunk (Takahashi et al., 2015). The aggressive encounter and all further experiments were terminated once (i) the partner mouse showed severe bleeding or ataxia, or (ii) the aggressor mice showed abnormal attack bites on any other body part. Partner mice fulfilling criteria (i) were euthanized. Aggressor mice fulfilling criteria (ii) were not used in any further experiments. If any aggressive sign (sideways threat, tail rattle, pursuit, and attack bite) was shown by the partner mouse, all further experiments with the partner mouse, aggressor mouse, and observer mouse were terminated.

### Overview

In total, 527 male C57BL/6J mice (CLEA Japan, Inc.), 49 female C57BL/6J mice (CLEA Japan, Inc.), and 33 male Slc:ICR mice (Japan SLC, Inc.; retired from used for breeding) were used in this study. In CLEA Japan, nursing females were individually housed (CL-0103-2; 165 mm × 234 mm × 118 mm), while pups were separated on P21 according to gender and housed ≤15 mice per cage (CL-0104-2; 206 mm × 317 mm × 125 mm). Pups were re-arranged on P28 according to their weights and housed ≤13 mice per cage (CL-0104-2). Mice were shipped in boxes each with 10–30 mice to the OIST Animal Facility. In the OIST Animal Facility, mice were housed in 380 mm × 180 mm × 160 mm transparent holding cages (Sealsafe Plus Mouse DGM–Digital Ready IVC; Tecniplast Inc., QC, Canada) bedded with 100% pulp (FUJ9298101; Oriental Yeast Co., Ltd., Tokyo, Japan) under a 12-h dark/light cycle (350-lux white light) at a controlled temperature of 22.7–22.9°C, humidity of 51–53%, and differential pressure of −14 to −8 Pa with food and water available *ad libitum*. Circadian time (CT) is defined to start at mid-light period and described in a 24-h format, i.e., light off at CT 6:00.

The experimenter and caretakers wore laboratory jumpsuits, lab boots, latex gloves, face masks, and hair nets when handling mice and performing experiments. Handling of mice during the dark cycle was done under dim red light and mice were transported in a lightproof carrier within the animal facility. For mice in experimental and control groups tested on the same dates, the testing order was alternated. Surfaces of experimental apparatuses were wiped with 70% ethanol in water and dry paper tissues after testing each mouse to remove olfactory cues. Each mouse was only used for one behavioral test (in total 4 records with intervals of 6–21 days) to avoid confounded results due to cross-testing and to minimize measurement effects on its psychological development (Krishnan et al., 2007).

### Pre-defeated Period (Day-21 to Day 0)

To establish partnerships between mice, a male C57BL/6J mouse (focal mouse; 8 weeks) was pair-housed with another male C57BL/6J mouse (partner mouse; 8 weeks) for 3 weeks (Day-21 to Day 0, with exposure to witnessing stress on Day 0). The partner was initially marked by ear punching. The holding cage was replaced once per week, with the last change 3 days before the defeated event (Day-3).

To establish the territory of an aggressor mouse in its homecage, an Slc:ICR mouse (aggressor mouse; ≥20 weeks) was pair-housed with a female C57BL/6J mouse (female mouse; 16 weeks) for 3 weeks (Day-21 to Day 0). The holding cage was replaced with a clean one once a week, with the last change 1 week before the defeated event (Day-7).

Aggression level of aggressors was screened on Days −5, −3, −1 through intruder encounters (Miczek and O’Donnell, 1978) toward different screening mice to determine appropriate aggressors to be used for exposure to witnessing stress on Day 0. Aggression screening was carried out in the behavior testing room at 22.4–23.0°C, 53–58% humidity, −4 to −3 Pa differential pressure, and 57.1 dB(C) ambient noise level during the light period (CT 4:00–6:00) with 350-lux white light. After the female and pups with the aggressor were taken out of their homecage and kept in a clean holding cage in the behavior testing room, a 3-min aggression screening was started after a male C57BL/6J mouse (screening mouse; 10 weeks) was brought into the homecage of the aggressor, followed by covering the cage with a transparent acrylic lid. During screening, the aggressor freely interacted with the screening mouse. The aggressor was brought back to the holding room after the screening mouse was taken away from the aggressor’s homecage and the female and pups were brought back to its homecage right after screening. Aggressors were selected for exposure to witnessing stress on Day 0 if they showed biting attacks on all of these screening days and the latencies to the initial bites on Day-3 and Day-1 were less than 20 s.

### Exposure to Witnessing Stress (Day 0)

The following experimental assay emotionally introduced an acute defeated experience in mice through a social process. The setup was the aggressor’s homecage, divided into an 80 mm × 180 mm auditorium zone and a 300 mm × 180 mm battle arena by the insertion of a stainless-steel mash with 8 mm × 8 mm lattices. The cage was covered with a transparent acrylic lid. The behavioral procedure was carried out in the behavior testing room during CT 4:00–6:00, with 3–5 experiments done in parallel.

After the female and pups with the aggressor were taken out of their homecage, a divider was inserted into the aggressor’s homecage, allowing the aggressor to freely behave in the battle arena, but not to enter the auditorium zone. A 5-min aggression encounter session started after the focal mouse was brought to the auditorium zone and its partner to the battle arena. Tail rattling counts of the focal mouse during aggressive encounter were recorded by experimenter. The aggressive encounter session was followed by a 5-min stress infiltration session, in which the partner was brought to the focal mouse in the auditorium zone, while the aggressor remained in the battle arena. Right after the stress infiltration session, both focal mouse and its partner were brought back to their homecage in the behavior testing room for a 10-min resting period. The procedure was repeated five times with different aggressors. During each resting session, the aggressor stayed in its homecage without the divider before its next intruder encounter. Each aggressor had 3–5 encounters with resting periods of 10–30 min. After the 5th aggression encounter session, the focal mouse was placed back in its homecage where the nest had been razed, and brought back to the holding room. Partners from different pairs were brought to a new holding cage and housed in groups of 3–5 per cage. Right after the last intruder encounter for each aggressor, the female and pups were brought back to the homecage and returned to the holding room together with the aggressor.

### Post-defeated Period (Day 0 to Day 28)

To investigate the behavior of focal mice after exposure to witnessing stress (now called ParObsIso mice, Figure 2A), they were housed individually for 4 weeks after the procedure (Day 0–Day 28). No environmental enrichment was provided, except to the ParObsIsoEE mice, and the holding cage was not changed during social isolation.

### Control Experiments

To differentiate behavioral consequences of the emotionally defeated experience from consequences of social isolation, a control group of mice had their partners taken away and their nests razed during body weighing on Day 0 without exposure to witnessing stress (xScenIso mice, Figure 1B).

To examine the potential reversal effects of social support on the emotionally defeated experience, a control group of mice was kept pair-housed with their attacked partners after exposure to witnessing stress (ParObsParPH mice, Figure 9A).

To characterize potential reversal effects through environmental factors besides social factors, a control group of mice was housed individually with environmental enrichment, provided with a pair of InnoDome™ and InnoWheel™ (Bio-Serv, Inc., Flemington, NJ, United States) and a Gummy Bone (Petite, Green; Bio-Serv, Inc.), after exposure to witnessing stress (ParObsIsoEE mice, Figure 9B).

To demonstrate predictive validity of potential treatment on stress by an antidepressant, a control group of mice was intraperitoneally injected with fluoxetine (2 μl/g of 10 mg/ml fluoxetine hydrochloride dissolved in saline, i.e., 20 mg/kg; F132-50 MG; Sigma-Aldrich, Inc., Saint Louis, MO, United States) once per day at CT 1:00–2:00 after exposure to witnessing stress (ParObsIsoFLX mice, Figure 9C).

To further test the critical component of social relationship in the potential social support reversal, a control group of mice was kept pair-housed but with a stranger mouse after exposure to witnessing stress (ParObsStrPH mice, Figure 11A).

To test the influence of social relationship on the emotionally defeated experience, a control group of mice witnessed the defeated events toward stranger mice of the same strain, gender, and age instead (StrObsIso mice, Figure 11B). In each iteration of the aggression encounter, stress infiltration, and resting period, a different stranger mouse was presented.

To identify anxiety-like spontaneous behaviors putatively induced by somatic uncertainty, a group of mice was initially sedated with 3%v/v isoflurane in oxygen and then intraperitoneally injected with caffeine (20 μl/g of 0.75 mg/ml anhydrous caffeine dissolved in saline, i.e., 15 mg/kg; 06712-55; Nacalai Tesque, Inc., Kyoto, Japan). Recording of spontaneous behaviors were started 30 min after the injections.

To identify anxiety-like spontaneous behaviors putatively induced by cognitive uncertainty, a group of mice was initially sedated with 3%v/v isoflurane in oxygen and then intraperitoneally injected with 20 μl/g saline. The mice received a series of foot shocks (1 mA for 1 s, 6 times in 5 min, i.e., once every 50 s of which the first shock started at 49 s after placed in the semi-transparent chamber in a soundproof box with 20-lux white fluorescent lamp illumination and ventilators; single chamber system; O’Hara & Co., Ltd., Tokyo, Japan) 25 min after the injections. Recording of spontaneous behaviors were started 30 min after the injections.

To identify non-treated-like spontaneous behaviors, a control group of mice was initially sedated with 3%v/v isoflurane in oxygen and then intraperitoneally injected with 20 μl/g saline. Recording of spontaneous behaviors were started 30 min after the injections.

### Body Mass and Nest Wall Height

In the holding room, body masses of all individuals were recorded on Days −7, 0, 1, 7, 28, while the heights of nest walls built by each individual were recorded on Days 1, 7, 28. The height of the nest wall was measured with 5-mm resolution using a transparent acrylic ruler, while the mouse was weighed with 10-mg resolution on a balance. Mice were placed back in their homecages right after recording.

### Light-Dark Box Test

The light-dark box test is an experimental assay to measure anxiety in rodents (Crawley and Goodwin, 1980), designed to evaluate their natural aversion to brightly lit areas against their temptation to explore. The light-dark box setup consisted of two connected 200 mm × 200 mm × 250 mm non-transparent PVC boxes, separated by a wall with a 50 mm × 30 mm door. The boxes were covered with lids with white and infrared LED light illumination for the light and dark areas, respectively, and CCD cameras in the centers (4-chamber system; O’Hara & Co., Ltd., Tokyo, Japan). The floors of the boxes were white, while the walls of the boxes were white for the light area and black for the dark area. Uniform illumination in the light area was 550 lux. Behavioral tests were carried out on Days −7, 1, 7, and 28 in the behavior testing room at 22.7–23.0°C, 51–54% humidity, −11 to −9 Pa differential pressure, and 53.6 dB(C) ambient noise level during dark period (CT 6:00–8:00).

After habituation for 10 min individually in the homecage in the behavior testing room in darkness, the focal mouse was transferred to the dark area through a 50 mm × 50 mm side door. A 5-min behavior record was started right after the side door of dark area was closed and the door between light and dark areas was opened. Locomotion was recorded 2-dimensionally at 15 Hz from top-view with CCD video cameras. Right after recording, the mouse was returned to its homecage, and brought back to the holding room.

### Elevated Plus-Maze Test

The elevated plus-maze test is an experimental assay to measure anxiety in rodents (Pellow et al., 1985), designed to evaluate their natural fear of falling and exposure against their temptation to explore. The elevated plus-maze setup consisted of a gray PVC platform raised 500 mm above the ground (single maze system; O’Hara & Co., Ltd.). The platform was composed of a 50 mm × 50 mm square central platform, two opposing 50 mm × 250 mm open arms, and two opposing 50 mm × 250 mm closed arms with 150-mm semi-transparent walls. Each of the two open arms emanated at 90° to each of the two closed arms, and vice versa. The apparatus was installed in a soundproof box with white fluorescent lamp illumination (20 lux) and ventilators. Behavioral tests were carried out on Days −7, 1, 7, 28 in the behavior testing room at 22.8–23.0°C, 53–56% humidity, −13 to −11 Pa differential pressure, and 52.1 dB(C) ambient noise level during dark period (CT 8:00–10:00).

After habituation for 10 min individually in the homecage in the behavior testing room in darkness, the focal mouse was brought to the central platform of the elevated plus-maze, facing the open arm on the opposite side from the door of the soundproof box. A 5-min behavior recording was started right after the door of the soundproof box was closed. Locomotion was recorded 2-dimensionally at 15 Hz from top-view with a CCD video camera installed above the center of the central platform. Delineated entrances to open and closed arms were defined at 50 mm from the center of the central platform. Right after recording, the mouse was placed back in its homecage, and brought back to the holding room.

### Open Field Test

The open field test is an experimental assay to measure anxiety in rodents (Hall and Ballachey, 1932), designed to evaluate their spontaneous activity under a gradient of spatial uncertainty (high in the field center and low along the walls and at the corners of the field). The open field setup consisted of a 400 mm × 400 mm × 300 mm non-transparent gray PVC box with no cover, installed in a soundproof box with white LED light illumination and ventilators (2-chamber system; O’Hara & Co., Ltd.). Behavioral tests were carried out on Days −7, 1, 7, 28 in the behavior testing room at 22.8–23.0°C, 53–56% humidity, −13 to −11 Pa differential pressure, and 56.7 dB(C) ambient noise level during dark period (CT 8:00–10:00).

After habituation for 10 min individually in the homecage in the behavior testing room in darkness, the focal mouse was brought to the center of the open field arena under 20-lux uniform illumination, facing the wall on the opposite side from the door of the soundproof box. A 5-min behavior recording was started right after the door of the soundproof box was closed. Locomotion was recorded 2-dimensionally at 15 Hz from top-view with a CCD video camera installed above the center of the open field arena. Vertical activity of exploratory rearing behavior was recorded by the blocking of invisible infrared beams created and detected by photocell emitters and receptors, respectively, positioned 60 mm high on the walls of the open field box. A delineated center region was defined as the central 220 mm × 220 mm area. Right after recording, the mouse was placed back in its homecage, and returned to the holding room.

### Locomotor Activity Test

The locomotor activity test is an experimental assay to measure spontaneous activity of rodents in an environment without an experimentally designed stressor. The locomotor activity setup consisted of a 200 mm × 200 mm × 250 mm non-transparent covered PVC box with infrared LED illumination and a CCD camera in the center (the dark area of the light-dark box setup, while the door between the light and dark areas was closed and fixed). The floor of the box was embedded with bedding material from the homecage of the focal mouse, while the walls of the box were black. Behavioral test was carried out on Days −7, 1, 7, 28 in the behavior testing room at 22.7–23.0°C, 51–54% humidity, −11 to −9 Pa differential pressure, and 53.6 dB(C) ambient noise level during dark period (CT 6:00–8:00).

After habituation for 30 min individually in the behavior testing box, a 1-h behavior recording was started. The behavior testing box was not covered completely to allow air circulation. Locomotion was recorded 2-dimensionally at 15 Hz from top-view with the CCD video camera. Right after recording, the mouse was returned to its homecage, and brought back to the holding room.

### Active Social Contact Test

The active social contact test [also known as “social interaction test,” but to be distinguished with the one-session test using an open field with a social target freely behaving in the field (Arakawa et al., 2014) or the one-session test placing a social target-containing cylinder into the center of the testing subject’s homecage for social instigation (Tsuda and Ogawa, 2012)] is a 2-session experimental assay to measure social motivation in rodents (Berton et al., 2006). The setup consists of a 400 mm × 400 mm × 300 mm non-transparent gray PVC box with no cover, installed in a soundproof box with 20-lux white LED illumination and ventilators. A 60 mm × 100 mm × 300 mm stainless-steel chamber with wire grid sides was placed in the center of the wall on the opposite side from the door of the soundproof box. The wire grid had 8 mm × 8 mm lattices at a height of 10–60 mm from the bottom. An ultrasound microphone (CM16/CMPA; Avisoft Bioacoustics, Glienicke, Germany) with an acoustic recording system (UltraSoundGate; Avisoft Bioacoustics) was hung outside the chamber, 100 mm above the ground. Behavioral tests were carried out on Days −7, 1, 7, 28 in the behavior testing room at 22.8–23.0°C, 53–56% humidity, −13 to −11 Pa differential pressure, and 56.7 dB(C) ambient noise level during dark period (CT 8:00–10:00).

The social target used for active social contact tests was either a male or a female C57BL/6J mouse (18 weeks), pair-housed with a partner of the same strain, gender, and age for more than 2 weeks before the tests. The social target was adapted to the experimental protocol one day before the tests in the behavior testing room during dark period (CT 8:00–9:00): After habituation for 5 min individually in the homecage in the soundproof box under 20-lux uniform illumination, the social target was brought into the chamber in the open field arena under 20 lux uniform illumination. A male C57BL/6J mouse (11–16 weeks; from partners of xScenIso mice in previous experiment) was then brought to the open field arena for a 2.5-min spontaneous exploration and interaction with the social target. The social target was then brought back to its homecage in the soundproof box under 20-lux uniform light for a 5-min rest. The social interaction procedure was repeated with a different male C57BL/6J mouse right afterward. After the social target had interacted with four different mice, it was returned to its homecage and brought back to the holding room.

On testing days, after 10-min habituation individually in its homecage in the behavior testing room in darkness, the first session of the active social contact test started by placing the focal mouse at the center of the open field arena under 20-lux uniform light, facing the empty chamber. A 2.5-min behavior recording started right after the door of the soundproof box was closed. Locomotion was recorded 2-dimensionally at 15 Hz from top-view with a CCD video camera installed above the center of the open field arena. Ultrasonic vocalization was recorded at 250 kHz. In the second session of the active social contact test, which followed the first session, the social target was brought into the chamber. Another 2.5-min behavior recording started as soon as the door of the soundproof box was closed. Right afterward, the focal mouse was returned to its homecage and brought back to the holding room.

The focal mouse experienced active social contact tests with different social targets on different recording days (Days −7, 1, 7, 28), while different focal mice were tested with the same social target on the same recording day (5–10 records). The social target remained in its homecage in a soundproof box under 20-lux uniform illumination before and between each test. A delineated interaction zone was taken as the region within 80 mm of the edges of the chamber. Social approaches of the focal mouse poking its nose toward the social target were recorded manually using the event recording software, tanaMove ver0.09.^1^

### Partner-Revisiting Test

The partner-revisiting test is a memory-based experimental assay to measure social bonding in rodents [sharing similar concept of “familiar vs. novel social target recognition,” but to be distinguished with the three-chamber paradigm test (Nadler et al., 2004)]. The partner-revisiting setup was the uncovered homecage of the focal mouse, installed in a soundproof box with white LED illumination and ventilators (O’Hara & Co., Ltd., Tokyo, Japan). The long sides of the homecage were parallel to the door of soundproof box. The partner-revisiting test was carried out on Day 28 in the behavior testing room at 22.8–23.0°C, 53–56% humidity, −13 to −11 Pa differential pressure, and 56.7 dB(C) ambient noise level during light period (CT 4:00–6:00) with 350-lux light intensity.

The previously separated partner of the focal mouse, being a social target in the test, was initially sedated with 3%v/v isoflurane in oxygen, and then anesthetized by intraperitoneal (i.p.) injection of a mixture of medetomidine (domitor, 3%v/v in saline, 0.3 mg/kg), midazolam (dormicum, 8%v/v in saline, 4 mg/kg), and butorphanol (vetorphale, 10%v/v in saline, 5 mg/kg). Also, a stranger mouse (15 weeks; a separated partner of a ParObsIso or Buffered mouse for testing a xScenIso, StrObsIso, or xAggrExpIso mouse, and *vice versa*) was anesthetized as an alternative social target. Both anesthetized mice were kept on a heating pad at 34°C (B00O5X4LQ2; GEX Co., Ltd., Osaka, Japan) to maintain their body temperatures before the test.

The focal mouse was brought to a clean, uncovered holding cage in the soundproof box under 50-lux uniform illumination for 5-min habituation, while its homecage was placed in another soundproof box under 50-lux uniform light. During habituation of the focal mouse, the anesthetized social targets were injected with atipamezole hydrochloride (antisedan; 6%v/v in saline for 0.3 mg/kg, i.p.) to induce recovery from anesthesia. During the waking-up period, the social targets were still immobilized and not able to actively interact with the focal mouse during the following recording but showed enough social cues to be attractive for the focal mouse. The immobilized social targets were then placed in the homecage of the focal mouse with their nose pointing toward the center of the short side of the wall (10 mm of nose-to-wall distance) with their bellies facing the door of the soundproof box. After habituation, the focal mouse was brought to the center of its homecage, facing the long side of the homecage wall on the opposite side from the door of soundproof box. A 10-min behavior record started right after the door of the soundproof box was closed. Locomotion was recorded 2-dimensionally at 15 Hz from top-view with a CCD video camera installed above the center of the homecage. Right after recording, social targets were taken out of the focal mouse’s homecage and the focal mouse was brought back to the holding room.

Social contacts including sniffing, allogrooming, and pushing of the focal mouse toward each of the social targets were recorded manually using the event recording software, tanaMove ver0.09 (see text footnote 1).

### Baseline Plasma Corticosterone Concentration Test

The baseline plasma corticosterone (CORT) concentration test is a competitive-inhibition enzyme-linked immunosorbent assay (ELISA) to measure physiological stress level in rodents, designed to quantitatively determinate CORT concentrations in blood plasma. The sample collection was carried out on Days −7, 1, 7, 28 in the behavior testing room at 22.4–23.0°C, 53–58% humidity, −4 to −3 Pa differential pressure, and 57.1 dB(C) ambient noise level during CT 4:00–6:00 with 350-lux white light.

After habituation for 30 min individually in the homecage in the behavior testing room, the mouse was initially sedated with 3%v/v isoflurane in oxygen. Six drops of blood from the facial vein pricked by a 18G needle were collected in a EDTA-lined tube [K2 EDTA (K2E) Plus Blood Collection Tubes, BD Vacutainer; Becton, Dickinson and Company (BD), Franklin Lakes, NJ, United States] and kept on ice. Right after collection, the mouse was returned to its homecage, and brought back to the holding room. Whole blood samples were then centrifuged (MX-300; Tomy Seiko Co., Ltd., Tokyo, Japan) at 3,000 rpm for 15 min at 4°C. Plasma supernatant was decanted and kept at −80°C until the measurement on Day 29.

Corticosterone concentrations in blood plasma were tested with Mouse Corticosterone (CORT) ELISA Kit (MBS703441, 96-Strip-Wells; MyBioSource, Inc., San Diego, United States; stored at 4°C before use) on Day 29. All reagents [assay plate (96 wells, pre-coated with goat-anti-rabbit antibody), standards (0, 0.1, 0.4, 1.6, 5, and 20 ng/ml of CORT), rabbit-anti-CORT antibody, HRP-conjugated CORT, concentrated wash buffer (20x phosphate-buffered saline (PBS)), 3,3′,5,5′-tetramethylbenzidine (TMB) color developing agent (substrates A and B), and TMB stop solution] and samples were brought to room temperature for 30 min before use. Collected plasma samples after thawing were centrifuged again (MX-300; Tomy Seiko Co.) at 3,000 rpm for 15 min at 4°C. 20 μl of standard or sample was added per well, assayed in duplicate, with blank wells set without any solution. After 20 μl of HRP-conjugated CORT was added to each well except to the blank wells, 20 μl of rabbit-anti-CORT antibody was added to each well and mixed. After incubation for 1 h at 37°C, each well was aspirated and washed, repeated for three times, by filling each well with 200 μl of wash buffer (diluted to 1x PBS) using a squirt bottle, standing for 10 s, and completely removing liquid at each step. After the last wash and the removal of any remaining wash buffer by decanting, the plate was inverted and blotted against clean paper towels. After TMB color developing agent (20 μl of substrate A and 20 μl of substrate B) was added to each well, mixed, and incubated for 15 min at 37°C in dark, 20 μl of TMB stop solution was added to each well and mixed by gently tapping the plate. The optical density (O.D.) of each well was determined, within 10 min, using a microplate reader (Multiskan GO; Thermo Fisher Scientific, Inc., Waltham, MA, United States) measuring absorbance at 450 nm, with correction wavelength set at 600–630 nm.

Corticosterone concentrations were calculated from the O.D. results using custom scripts written in MATLAB R2015b (MathWorks). The duplicate O.D. readings for each standard and sample was averaged and subtracted the average O.D. of the blanks, *X* = < *O.D*. > − < *O.D*. > _*blank*_. A standard curve was determined by a four parameter logistic (4PL) regression fitting the equation $\rho_{C\,O\,R\,T}\,\left(X_{s\,t\,a\,n\,d\,a\,r\,d}\right)=d+\frac{a-d}{1+{\left(\frac{X_{s\,t\,a\,n\,d\,a\,r\,d}}{c}\right)}^{b}}$, where ρ_*CORT*_ is the CORT concentration, *a* is the minimum asymptote, *b* is the Hill’s slope, *c* is the inflection point, and *d* is the maximum asymptote. CORT concentrations of the samples were calculated from the fitted 4PL equation with respected to *X*_*sample*_.

### *Ex vivo* Diffusion Tensor Imaging

*Ex vivo* DTI is a magnetic resonance imaging (MRI) technique to determinate structural information about tissues (Basser et al., 1994), designed to measure the restricted diffusion of water in tissue. The sample collection was carried out on Day 28 in the necropsy room at 22.4–22.5°C, 53–54 % humidity, and 10–12 Pa differential pressure during CT 4:00–6:00 with 750-lux white light.

After mice were brought individually in their homecages to the necropsy room, they were initially sedated with 3%v/v isoflurane in oxygen, then deeply anesthetized with a ketamine-xylazine mixture (>30 μl/g body weight of 100 mg/ml ketamine and 20 mg/ml xylazine), and perfused transcardially. The perfusates, in a two-step procedure, were (i) 20 ml of ice cold 1x phosphate-buffered saline (PBS), and (ii) 20 ml of ice cold 4% paraformaldehyde, 0.2% sodium meta-periodate, and 1.4% lysine in PBS. Mouse skull including the brain was removed and stored in the perfusate (ii) at 4°C for 2 weeks. Each skull with the brain was then transferred into 2 mM gadolinium with diethylenetriaminepentaacetic acid (Gd-DTPA) and 0.5% azide in PBS for 2 weeks.

Isolated fixed brains within the skulls were positioned in an acrylic tube filled with fluorinert (Sumitomo 3M Ltd., Tokyo, Japan) to minimize the signal intensity attributable to the medium surrounding the brain during MRI scanning. All MRI was performed with an 11.7-T MRI system (BioSpec 117/11; Bruker Biospec, Ettlingen, Germany) using ParaVision 6.0.1 software (Bruker Biospec, Ettlingen, Germany) for data acquisition. The inner diameter of the integrated transmitting and receiving coil (Bruker Biospec, Ettlingen, Germany) was 35 mm for the *ex vivo* MRI. DTI data were acquired by using a 3-D diffusion-weighted spin-echo imaging sequence, with repetition time (TR) = 267 ms, echo time (TE) = 18.5 ms, *b*-value = 2,000 s/mm^2^, and 30 non-collinear directions. Five T2-weighted measurements were acquired together with DTI, for one every six diffusion measurements. The acquisition matrix was 216 × 216 × 168 over a 27.0 mm × 27.0 mm × 21.0 mm field of view, resulting in a native isotropic image resolution of 125 μm. Total acquisition time was 96 h.

Magnetic resonance imaging data was processed using custom scripts written in MATLAB R2015b (MathWorks). All 30 DTI and 5 T2 3-D images were masked by thresholding at the half of mean values of diffusion weights for each voxel and omiting clusters smaller than 10 voxels. After diffusion tensor of each voxel was estimated by solving the Stejskal-Tanner equation through linear regression (Hrabe et al., 2007), the three eigenvalues (λ_1_, λ_2_, and λ_3_) with respect to the three axes of the diffusion ellipsoid (the longest, middle, and shortest axes, respectively) were calculated by eigenvalue decomposition of the diffusion tensor. Four focused DTI-based measures (Mori, 2007) are the mean diffusivity (MD) that represents membrane density


$$M\,D=<\lambda >=\frac{\lambda_{1}+\lambda_{2}+\lambda_{3}}{3},$$

axial diffusivity (AD) that represents neurite organization


$$A\,D=\lambda_{1},$$

radial diffusivity (RD) that represents myelination


$$M\,D=\frac{\lambda_{2}+\lambda_{3}}{2},$$

and fractional anisotropy (FA) that represents average microstructural integrity


F⁢A=3×[(λ1-⟨λ⟩)2+(λ2-⟨λ⟩)2+(λ3-⟨λ⟩)2]2×(λ12+λ22+λ32).

After the registration of these 3-D DTI-based brain maps to a template brain atlas (DSURQE Atlas),^2^ mean values of these DTI-based quantities were identified in a total of 244 brain regions (Figure 2) for each individual. Based on the FA maps, DTI-based long-range fiber tracking from a focal seed region (Figures 3E,F) was calculated with two samplings, distance of forward fiber in one step = 50 μm, and thresholds of minimal fiber length = 750 μm, maximal fiber length = 75,000 μm, maximal fiber deviation angle = 57.3°, and minimal FA for keeping tracking = 0.4.

### Video Data Processing

Video image data was processed using custom scripts written in MATLAB R2015b (MathWorks). Each video frame from a recorded AVI video file was read as a 2-dimensional matrix with an 8-bit gray scale. Each of these matrices was then divided by a background matrix read from a TIF image file of the background taken before bringing the test mouse to the setup. The centroid of the area with non-one values in each matrix ratio was taken as the position of the mouse at this specific time point. Speed was calculated as the distance between temporally adjacent positions multiplied by 15 (15-Hz recording). Freezing periods were sorted out if the area of the mouse body between temporally adjacent frames was less than 20 mm^2^. Nose and tailbase position of each frame in the active social contact tests was identified by DeepLabCut (MobileNetV2-0.35, 1030000 iterations) (Mathis et al., 2018).

### Audio Data Processing

Audio signal data was processed with custom scripts written in MATLAB R2015b (MathWorks). Each recorded WAV audio file was read and transformed into a spectrogram using fast Fourier transform with non-overlapping 0.4-ms time windows. To identify the time segments with ultrasonic vocalization signals, recordings were thresholded at a power spectral density (PSD) ≥−75 dB/Hz, and time segments with averaged PSD between 0 and 50 kHz higher than that between 50 and 120 kHz were removed. The duration of remaining time segments was calculated.

### Statistical Analysis

Numerical data were analyzed with custom scripts written in MATLAB R2015b (MathWorks). Statistical significance of the difference between 2 mean values was estimated with Tukey’s range test following the analysis of variance with the factor of experimental paradigms. Statistical significance of the difference between 2 median values (vocalization analysis; Figure 8E) was estimated using one-tailed Mann–Whitney *U*-test.

To capture fine-scale behavioral details of location within the light-dark box and the elevated plus-maze (Figures 4, 5), we computed T(x), the cumulative probability of finding position ≤x, for each individual (light traces) for all measured locations (a collection of locations from all mice for the statistics). We then show the average across the control group (bold blue trace) and the ParObsIso group (bold red trace). We compared the averages of each group with a two-tailed, two-sample Student’s *t*-test and plot the resulting *p*-values, presented as -log(p), the negative logarithm of *p*-values. We also show the box plot (the minimum, lower quartile, median, upper quartile, and maximum) of -log(p) values collapsed across all measured locations. To capture the fine-scale behavioral details of speed, we followed a similar procedure as above, but with U(v), the cumulative distribution function of finding speed ≤v.

To estimate local likelihoods of caffeine-injected, foot-shocked, and non-treated behavior in the light-dark box or elevated plus-maze tests for any given 4-dimensional behavioral states described by the position, speed, velocity along the stressor axis, and acceleration strength, we trained a deterministic 3-layer feedforward network with hidden layer sizes of 26, 30, and 24 units, respectively, using log-sigmoid transfer functions. For pattern recognition, each network was trained by using the scaled conjugate gradient method to minimize cross-entropy to obtain reliable classifiers, with a random data division of 80% for training and 20% for testing. Training of updating weights and biases terminated when one of the following condition was matched: (1) reaching 1,000 iterations, (2) obtaining a perfect data fitting [i.e., the mean squared error (MSE) equaled to zero], (3) having the error rate continuously increasing for more than 6 epochs, (4) showing the gradient of MSE less than 10^–7^, and (5) receiving the training gain larger than 10^10^. The global likelihoods of a recorded mouse to be caffeine-injected-like, foot-shocked-like, and non-treated-like were calculated by taking the average of local likelihoods of each experimental type estimated by the corresponding trained network.

To evaluate the uncertainty of the percentage for each tail rattle count (Figure 13A), we created 10,000 bootstrapped data sets where each sample was randomly picked with replacement from the original data set. Each bootstrapped data set had the same sample size as the original data set. The standard error was taken as the standard deviation of the bootstrapped percentages for a tail rattle count. A similar procedure was carried out to evaluate the standard error of mean for the percentage of time spent in each behavior in the partner-revisiting test (Figure 14B), where each sample of the bootstrapped data sets was a set of the percentages of the three classified behaviors (partner concern, stranger concern, and non-social activity/sniffing at social targets) from a mouse record. Standard errors of means for other results were estimated with the formula $\frac{\sigma }{\sqrt{n}}$, where σ is the sample standard deviation and *n* is the sample size.

## Data Availability Statement

The raw data supporting the conclusions of this article will be made available by the authors, without undue reservation.

## Ethics Statement

The animal study was reviewed and approved by the OIST IACUC in the AAALAC-accredited facility.

## Author Contributions

RL conceived the study, designed the research, performed the experiments, analyzed the data, interpreted the results, wrote the original draft, and revised the manuscript. GS advised on data analysis and revised the manuscript. BK advised on research design, revised the manuscript, and supervision. All authors contributed to the article and approved the submitted version.

## Conflict of Interest

The authors declare that the research was conducted in the absence of any commercial or financial relationships that could be construed as a potential conflict of interest.

## Publisher’s Note

All claims expressed in this article are solely those of the authors and do not necessarily represent those of their affiliated organizations, or those of the publisher, the editors and the reviewers. Any product that may be evaluated in this article, or claim that may be made by its manufacturer, is not guaranteed or endorsed by the publisher.

## Footnotes

1
http://www.mgrl-lab.jp/tanaMove.html

2
https://wiki.mouseimaging.ca/display/MICePub/Mouse+Brain+Atlases

## Supplementary Material

Supplementary Figure 1 Comparison of behavioral characteristics in high-dimensional state-space indicates chronic somatic and cognitive anxiety developed in stress incubation. **(A)** Foot-shocked mice displayed less time spent in the light area and slower locomotion compared with the saline-injected and caffeine-injected mice in the light-dark box test (*n*_*saline*_ = 5, *n*_*caffeine*_ = 5, *n*_*footshock*_ = 5). **(B)** Caffeine-injected mice displayed less time spent in the opened arms compared with the saline-injected and foot-shocked mice in the elevated plus-maze test (*n*_*saline*_ = 5, *n*_*caffeine*_ = 5, *n*_*footshock*_ = 5). **(C)** In the light-dark box test, xScenIso mice stably showed non-treated-like behavioral characteristics after separated with its pair-housed partners, while ParObsIso mice increased their caffeine-injected-like behavioral characteristics in the corresponding period (*n*_*saline*_ = 5, *n*_*caffeine*_ = 5, *n*_*footshock*_ = 5). **(D)** In the elevated plus-maze test, xScenIso mice kept showing highest likelihood of behavioral characteristics as non-treated-like after separated with its pair-housed partners, while ParObsIso mice increased their foot-shocked-like behavioral characteristics in the corresponding period (*n*_*xScenIso*_ = 8, *n*_*ParObsIso*_ = 8). Note that comparison of behavioral characteristics in high-dimensional state-space was tested by Tukey’s range test.

Supplementary Figure 2 Evenly distributed nose-tailbase orientation of ParObsIso mice after trauma induction supports the reduction of social interest. Orientation angle of each frame were calculated from the tailbase-to-nose vector and the tailbase-to-wall-center (the wall of the chamber side) vector.

Supplementary Figure 3 Locomotor activity tests in control experiments agree with the conclusions of spontaneous behaviors given from the light-dark box tests. The results of ParObsIsoFLX mice.

Supplementary Figure 4 Elevated plus-maze tests in control experiments agree with the conclusions of spontaneous behaviors given from the light-dark box tests. **(A)** The results of ParObsParPH mice. **(B)** The results of xAggrExpIso mice.

Supplementary Video 1 Landscape in the 4-D state space of local likelihood of caffeine-injected behaviors in the light-dark box test.

Supplementary Video 2 Landscape in the 4-D state space of local likelihood of foot-shocked behaviors in the light-dark box test.

Supplementary Video 3 Landscape in the 4-D state space of local likelihood of control behaviors in the light-dark box test.

Supplementary Video 4 Landscape in the 4-D state space of local likelihood of caffeine-injected behaviors in the elevated plus-maze test.

Supplementary Video 5 Landscape in the 4-D state space of local likelihood of foot-shocked behaviors in the elevated plus-maze test.

Supplementary Video 6 Landscape in the 4-D state space of local likelihood of control behaviors in the elevated plus-maze test.

Supplementary Video 7 Social apathy is an observed behavioral characteristic of ParObsIso mice. Examples of 30-s recordings during the social session in the female stranger test on Day 1. First scene, xScenIso mouse; Second scene, ParObsIso mouse.

Supplementary Video 8 Illustration of behavioral characteristics that were specific to observer mice during exposure to witnessing stress when their partners were attacked. First scene, tail rattling during aggressive encounter; Second scene, tail rattling during aggressive encounter (4 × slower); Third scene, hiding under bedding material with the partner during resting. These behaviors were not observed if a stranger mouse got attacked.

Supplementary Video 9 Illustration of rebound reaction of a ParObsIso mouse to its previously pair-housed partner during the partner-revisiting test.
