# Supplementary figures and images for: Social Relationship as a Factor for the Development of Stress Incubation in Adult Mice
Source: Front Behav Neurosci. 2022 May 24;16:854486. doi: 10.3389/fnbeh.2022.854486 (PMC9172995; doi:10.3389/fnbeh.2022.854486)

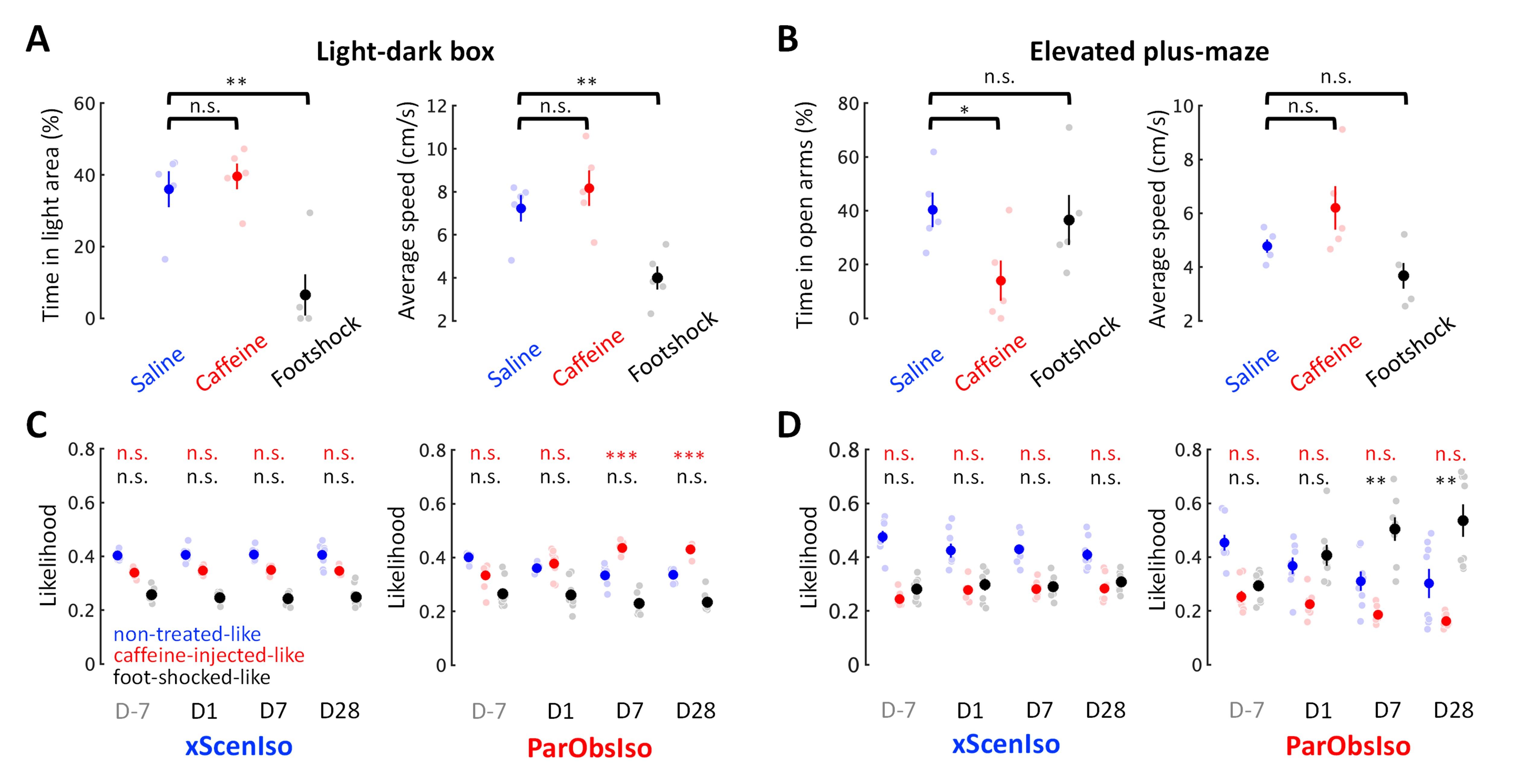

Supplement: Supplementary Figure 1 — Comparison of behavioral characteristics in high-dimensional state-space indicates chronic somatic and cognitive anxiety developed in stress incubation. (A) Foot-shocked mice displayed less time spent in the light area and slower locomotion compared with the saline-injected and caffeine-injected mice in the light-dark box test (nsaline = 5, ncaffeine = 5, nfootshock = 5). (B) Caffeine-injected mice displayed less time spent in the opened arms compared with the saline-injected and foot-shocked mice in the elevated plus-maze test (nsaline = 5, ncaffeine = 5, nfootshock = 5). (C) In the light-dark box test, xScenIso mice stably showed non-treated-like behavioral characteristics after separated with its pair-housed partners, while ParObsIso mice increased their caffeine-injected-like behavioral characteristics in the corresponding period (nsaline = 5, ncaffeine = 5, nfootshock = 5). (D) In the elevated plus-maze test, xScenIso mice kept showing highest likelihood of behavioral characteristics as non-treated-like after separated with its pair-housed partners, while ParObsIso mice increased their foot-shocked-like behavioral characteristics in the corresponding period (nxScenIso = 8, nParObsIso = 8). Note that comparison of behavioral characteristics in high-dimensional state-space was tested by Tukey’s range test. [file Image_1.jpg]

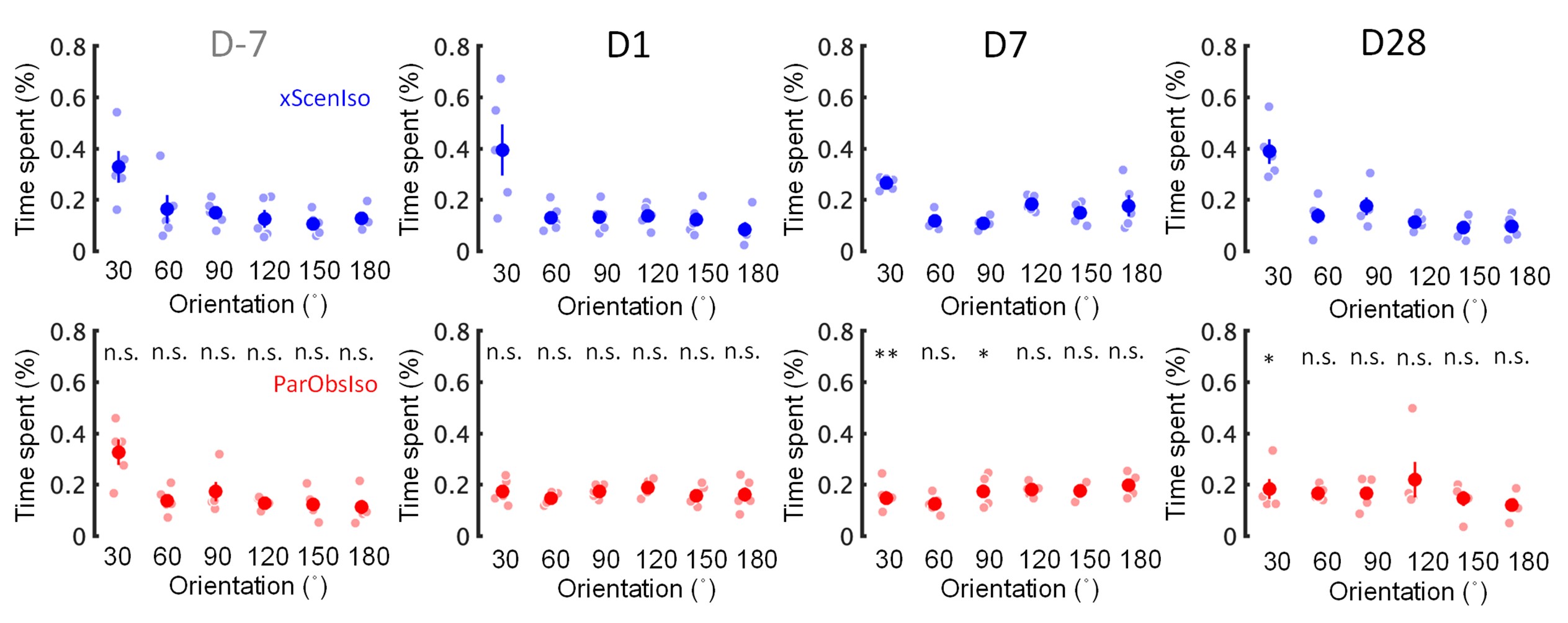

Supplement: Supplementary Figure 2 — Evenly distributed nose-tailbase orientation of ParObsIso mice after trauma induction supports the reduction of social interest. Orientation angle of each frame were calculated from the tailbase-to-nose vector and the tailbase-to-wall-center (the wall of the chamber side) vector. [file Image_2.jpg]

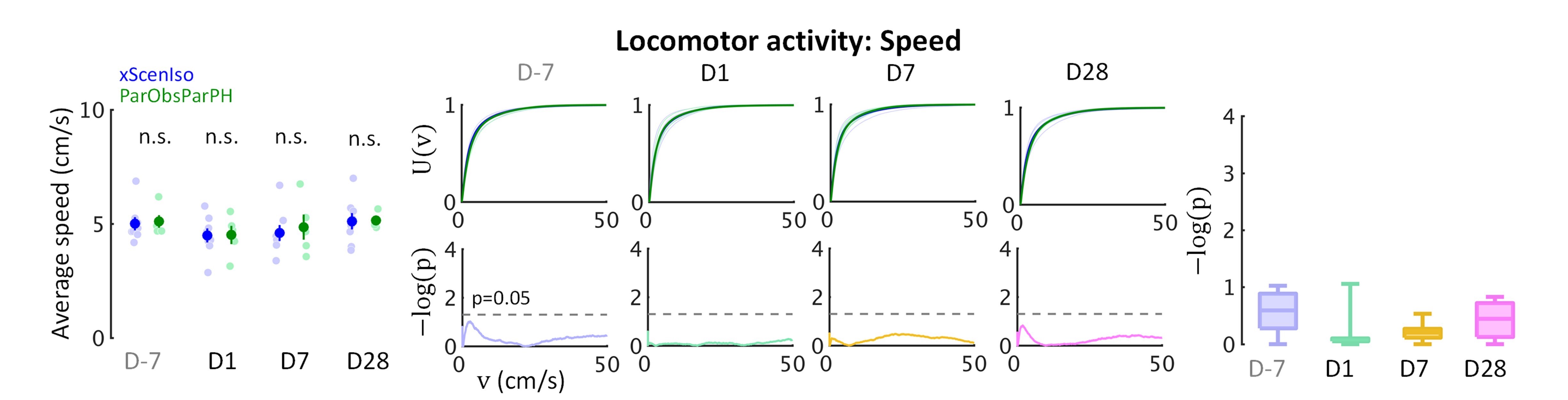

Supplement: Supplementary Figure 3 — Locomotor activity tests in control experiments agree with the conclusions of spontaneous behaviors given from the light-dark box tests. The results of ParObsIsoFLX mice. [file Image_3.jpg]

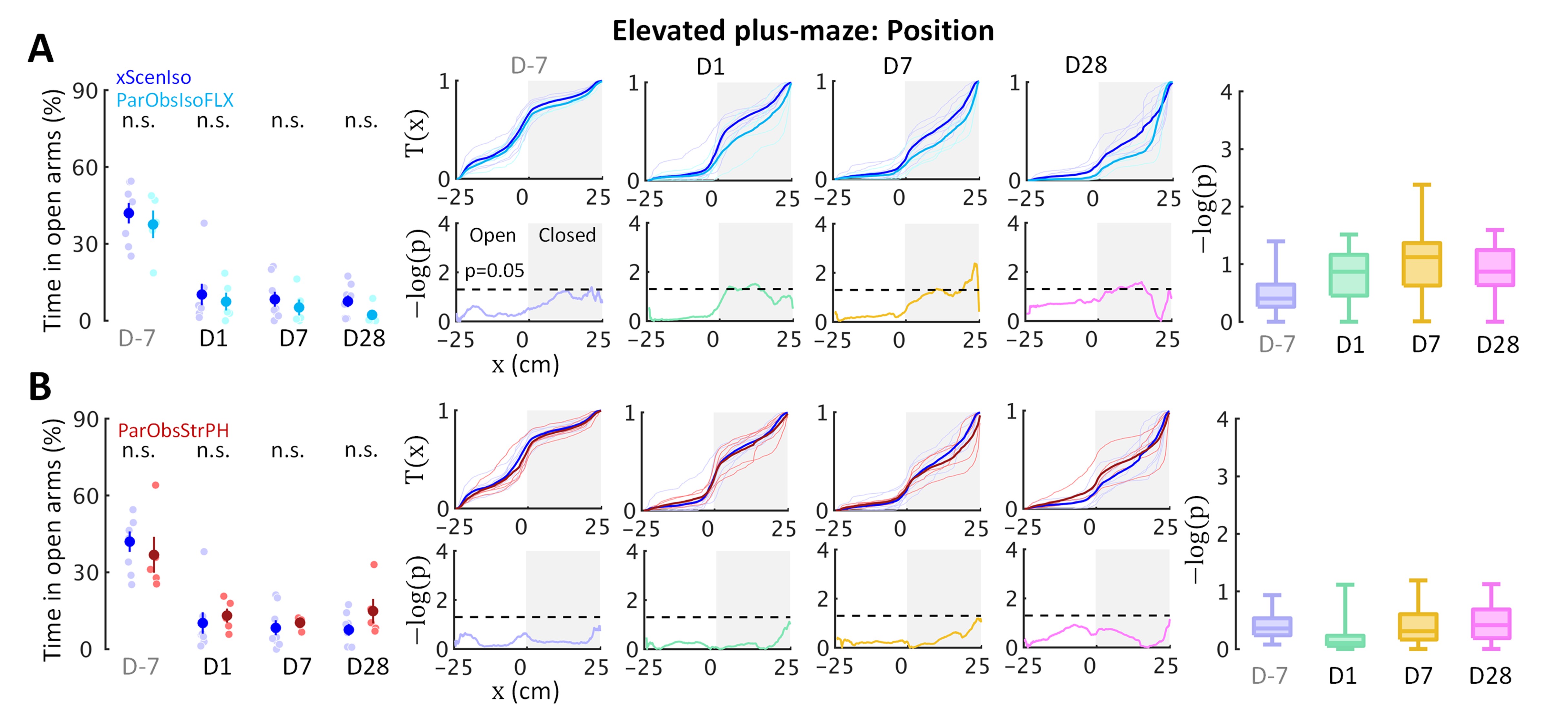

Supplement: Supplementary Figure 4 — Elevated plus-maze tests in control experiments agree with the conclusions of spontaneous behaviors given from the light-dark box tests. (A) The results of ParObsParPH mice. (B) The results of xAggrExpIso mice. [file Image_4.jpg]
